# MicroRNAs as a clue to overcome breast cancer treatment resistance

**DOI:** 10.1007/s10555-021-09992-0

**Published:** 2021-09-15

**Authors:** Iris Garrido-Cano, Birlipta Pattanayak, Anna Adam-Artigues, Ana Lameirinhas, Sandra Torres-Ruiz, Eduardo Tormo, Raimundo Cervera, Pilar Eroles

**Affiliations:** 1 INCLIVA Biomedical Research Institute, 46010 Valencia, Spain; 2 Center for Biomedical Network Research On Cancer, CIBERONC-ISCIII, 28029 Madrid, Spain; 3 Department of Physiology, University of Valencia, 46010 Valencia, Spain

**Keywords:** MicroRNAs, Resistance, Breast, Cancer

## Abstract

Breast cancer is the most frequent cancer in women worldwide. Despite the improvement in diagnosis and treatments, the rates of cancer relapse and resistance to therapies remain higher than desirable. Alterations in microRNAs have been linked to changes in critical processes related to cancer development and progression. Their involvement in resistance or sensitivity to breast cancer treatments has been documented by different *in vivo* and *in vitro* experiments. The most significant microRNAs implicated in modulating resistance to breast cancer therapies are summarized in this review. Resistance to therapy has been linked to cellular processes such as cell cycle, apoptosis, epithelial-to-mesenchymal transition, stemness phenotype, or receptor signaling pathways, and the role of microRNAs in their regulation has already been described. The modulation of specific microRNAs may modify treatment response and improve survival rates and cancer patients’ quality of life. As a result, a greater understanding of microRNAs, their targets, and the signaling pathways through which they act is needed. This information could be useful to design new therapeutic strategies, to reduce resistance to the available treatments, and to open the door to possible new clinical approaches.

## Introduction

Cancer is one of the major causes of death and morbidity worldwide, leading to numerous adverse socioeconomic effects. Particularly, breast cancer (BC) has been the most frequently diagnosed cancer in 2020, with 2.3 million new cases worldwide. Although lung cancer remains the leading cause of cancer death (18%), BC accounts for 6.9% of the total [1]. Resistance to treatments is currently one of the main challenges in the clinical management of BC patients. The latest generation of anti-BC agents has increased the specificity and effectiveness of the treatment. Despite this, the molecular mechanisms of drug resistance are often not fully understood and difficult to overcome. A deeper understanding of the processes involved in BC development, the mechanisms of action of therapies, and the causes of resistance may aid in modulating and improving the response to the current treatments.

Epigenetic mechanisms, and specifically microRNAs (miRNAs), can act as regulators of treatment response. Modulation of gene expression by miRNAs is the most important post-transcriptional regulatory mechanism. miRNAs regulate the expression of their target genes mostly through binding to the 3′ untranslated regions (3′UTR) from messenger RNAs (mRNAs), which leads to their degradation or the inhibition of their translation into protein. Remarkably, the role of miRNAs in the pathogenesis of cancer has been widely described [2–16]. miRNAs are known to be implicated in regulating the hallmarks of cancer such as cell proliferation, growth, apoptosis, invasion, metastasis, immune system scape, and altered metabolism [17]. Importantly, miRNAs can also modulate drug sensitivity/resistance by regulating genes involved in biological processes related to response to treatments.

Outstandingly, miRNAs show high stability, which makes them good candidates as non-invasive biomarkers for early diagnosis, follow-up, response prediction, and even as a possible treatment strategy for cancer patients. This promising scenario has led to extensive research about miRNAs in cancer. In the BC research field, most of the studies are focused on the miRNAs’ differential expression between cancer patients’ and healthy donors’ samples and their potential use in diagnosis, as well as in uncovering the cancer-related mechanisms behind miRNAs dysregulation. However, just a small percentage of the studies evaluate the influence of miRNAs in response to a particular treatment. Given the importance of identifying biomarkers for treatment response prediction and the critical function of miRNAs in the control of fundamental biological processes, the role of miRNAs in BC therapy response must be highlighted.

In this review, the available information about miRNAs that modulate the response to specific BC treatments and how they regulate several cellular processes is summarized. Deeply understanding of these processes could help to improve clinical approaches and to adapt therapies to individual patients in a process called precision medicine.

### miRNAs and cell cycle

**Fig. 1:**
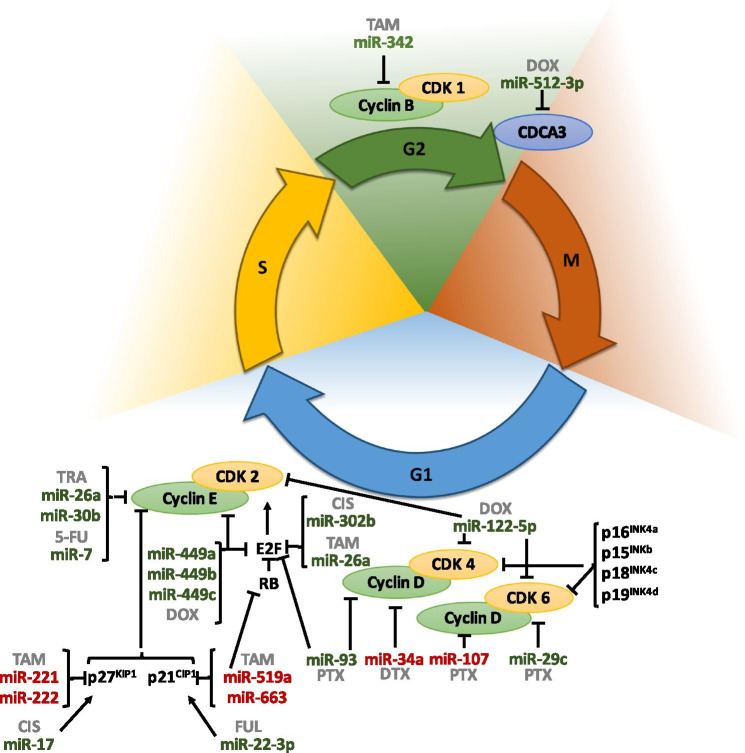
Schematic representation of miRNAs involved in drug resistance through regulating cell cycle. Arrows indicate activation and line with a perpendicular line at the end indicates inhibition. miRNAs increasing drug resistance are represented in red color, and miRNAs increasing drug sensitivity are represented in green color. CIS, cisplatin; DOX, doxorubicin; DTX, docetaxel; FUL, fulvestrant; PTX, paclitaxel; RAD, radiation; TAM, tamoxifen; TRA, trastuzumab; 5-FU, 5-fluorouracil

Cell cycle dysregulation is a recognized hallmark of cancer, and its aberrant activation has been related to poor prognosis and drug resistance. The cell cycle includes four sequential phases: G0/G1 (Gap 0/1), S (synthesis), G2 (Gap 2), and M (mitosis) that are regulated by cyclin-dependent kinases (CDKs). CDKs play an important role in controlling cell division, which leads to cellular growth. They are hyperactivated and dysregulated in several types of tumors [18–20]. Different miRNAs have been described to target genes involved in cell cycle regulation, leading to drug resistance or sensitivity (Table 1 and Fig. 1).

**Table.1:** MiRNAs and cell cycle (bold, direct targets; italicized, indirectly downregulated targets; italicized and *, indirectly upregulated targets)

MiRNAs	Drug	Targets	Reference
miR-7	5-Fluorouracil	**CCNE1**	[28]
miR-16	Trastuzumab Lapatinib	**FUBP1** **CCNJ**	[27]
miR-17	Cisplatin	**JAB1** *p27*^kip1^*	[37]
miR-22-3p	Fulvestrant	**FOXP1** **HDAC4** *p21*^cip1^*	[34]
miR-26a	Tamoxifen	**E2F7**	[31]
	Trastuzumab	**CCNE2**	[29]
miR-29c	Paclitaxel	**CDK6**	[38]
miR-30b	Trastuzumab	**CCNE2**	[29]
miR-34a	Docetaxel	**CCND1**	[21]
miR-93	Paclitaxel	**E2F1** **CCND1**	[22]
miR-107	Paclitaxel	**TPD52** *CCND1*	[24]
miR-122-5p	Doxorubicin	**CDK2** **CDK4** **CDK6**	[39]
miR-221/222	Tamoxifen	**p27**^**kip1**^	[36]
miR-223	Palbociclib	*EGF pathway*	[40]
miR-302b	Cisplatin	**E2F1**	[23]
miR-342	Tamoxifen	**GEMIN4** **BMP7** *CCNB1*	[25]
miR-449a miR-449b miR-449c	Doxorubicin	**CCNE2** **CDK2** **E2F**	[32]
miR-512-3p	Doxorubicin	**CDCA3**	[26]
miR-519a	Tamoxifen	**p21**^**CIP1**^ **RB1**	[33]
miR-663b	Tamoxifen	**TP73** TP53* p21*	[35]

On the one hand, several miRNAs have been shown to induce cell cycle arrest as a result of targeting cyclins. One of them is miR-34a, which was demonstrated to increase resistance to docetaxel (DTX) in luminal BC cells, probably through inhibition of cyclin D1 (*CCND1*) and B-cell lymphoma 2 (*BCL-2*), then inducing G1 arrest and blocking DTX effectiveness as a consequence [21]. miR-93 has also been linked to cell cycle arrest in the G1/S phase. Bao et al. [22] described that miR-93 expression was reduced in paclitaxel (PTX)-resistant BC samples compared to responder patients. E2F transcription factor 1 (*E2F1*) and *CCND1* were found to be direct targets of this miRNA. Their downregulation led to cell cycle arrest in G1 and enhanced apoptosis due to the inhibition of AKT phosphorylation (p-AKT), reduction of BCL-2, and increment of BCL-2-associated X, apoptosis regulator (BAX) expression levels, which could result in an enhancement of PTX sensitivity. *E2F1* has been also identified as a direct target gene of miR-302b, which increases sensitivity to cisplatin (CIS) [23]. The tumor protein D52 (*TPD52*) was identified as a direct target of miR-107, which was found to be upregulated in BC tumors compared to healthy breast samples. TPD52 downregulation leads to a decrease in CCND1 and PTX resistance [24]. Another study found downregulation of miR-342 in tamoxifen (TAM)-resistant BC cell lines and tumors. This miRNA directly targets Gem nuclear organelle-associated protein 4 (*GEMIN4*) and Bone morphogenetic protein 7 (*BMP7*), leading to indirect cyclin B1 (CCNB1) downregulation. Restoration of the expression of miR-342 increased BC cells’ sensitivity to TAM by enhancing apoptosis and cell cycle arrest [25]. Furthermore, the expression of miR-512-3p was found to be downregulated in triple-negative BC (TNBC). Its expression was related to cell cycle arrest, reduced proliferation, migration, and also with doxorubicin (DOX) sensitivity by directly targeting cell division cycle associated 3 (*CDCA3*), which is an oncogene that triggers mitotic entry [26]. Moreover, it was found that trastuzumab (TRA) and lapatinib (LAP) treatments in human epidermal growth factor receptor 2 positive (HER2 +) BC cells blocked phosphoinositide 3-kinase (PI3K) pathway resulting in high miR-16 levels. Far upstream element binding protein 1 (*FUBP1*) and Cyclin J (*CCNJ*) were identified as its direct targets, which when upregulated, promote proliferation. Consequently, miR-16 upregulation inhibited BC cell proliferation and correlated with good treatment response to anti-HER2 therapy [27].

Considering cyclin E, an important regulator of the G1/S transition, Yang et al. [28] showed that cyclin E1 (*CCNE1*) was a direct target of miR-7, whose reduced expression was related to 5-fluorouracil (5-FU) resistance. It was demonstrated that miR-7 overexpression could restore the sensitivity to 5-FU chemotherapy treatment. Besides, miR-30b and miR-26a have also been identified as TRA-response regulators by directly regulating cyclin E2 (*CCNE2*) and halting the cell cycle in G1 [29, 30]. Moreover, E2F transcription factor 7 (E2F7) was found upregulated in estrogen receptor-positive (ER +) BC, leading to TAM resistance. Liu et al. [31] described *E2F7* as a miR-26a direct target and this miRNA reversed TAM resistance. Furthermore, miR-449a, miR-449b, and miR-449c are implicated in cell cycle control and chemotherapy resistance. Tormo et al. [32] found its dysregulation in DOX-treated cells. Herein, miR-449a, b, and c reduced cell cycle regulators such as *CCNE2*, *E2F1, E2F3*, and CDK2, resulting in a cell cycle arrest in G0. Additionally, overexpression of miRs-449 in resistant TNBC cells was able to re-establish sensitivity to DOX.

In addition to the cyclin E regulation, miRNAs can also target p21^cip1^ and p27^kip1^, which are cyclin E inhibitors. On the one hand, miR-519a, miR-22-3p, and miR-663b have been described to downregulate p21^cip1^. miR-519 was found to be upregulated in TAM-resistant cells and correlated with poor prognosis and lower survival. It was shown to target three important cell cycle and PI3K/AKT pathway elements: *p21*^*cip1*^, Phosphatase and Tensin Homolog (*PTEN*), and RB transcriptional corepressor 1 (*RB1*) [33]. By contrast, miR-22-3p and miR-663b have the opposite effect as those miRNAs upregulate p21^cip1^ expression. miR-22-3p has a dual role in re-sensitizing fulvestrant (FUL)-resistant BC cells through a p21^cip1^-dependent mechanism, as it targets their transcriptional repressors forkhead box P1 (FOXP1) and Histone Deacetylase 4 (HDAC4), and also through p53 acetylation, leading to activation of p21^cip1^ [34]. Concerning miR-663b, Jiang et al. [35] described that it directly regulates the expression of TP73, which is a tumor suppressor protein that controls cell cycle and apoptosis through p53 trans-activation response genes and increases TAM resistance. miR-663b inhibition downregulated BCL-2 and upregulated cell cycle and apoptosis gene regulators such as *p53*, *p21*^*cip1*^, and *Bax.*

On the other hand, multiple miRNAs control p27^kip1^ levels. miR-221/222 were shown to directly target this gene. Miller et al. [36] found that their expression was related to resistance to TAM in HER2 + BC cells by targeting *p27*^*kip1*^. Besides this, Wang et al. [37] observed that miR-17 was downregulated in TNBC, acting as a tumor suppressor and indirectly regulating p27^kip1^ expression through its direct target c-Jun activation domain-binding protein-1 (*JAB1*). It was described that *JAB1* expression was related to proliferation, invasion, p27^kip1^ repression, and also with CIS resistance. Downregulation of *JAB1* resulted in higher p27^kip1^ levels, cell cycle arrest in G1, and a higher sensitivity to CIS.

Moreover, some other miRNAs have been shown to modulate drug resistance through targeting CDKs. One of them is miR-29c, which was downregulated in BC tissues compared to healthy tissues, being *CDK6* its direct target [38]. miR-29c overexpression decreased CDK6 level, inducing cell cycle arrest and PTX sensitivity. Furthermore, Zang et al. [39] analyzed the expression of miR‐122‐5p in DOX-resistant cells under resveratrol treatment. Results showed that miR‐122‐5p was upregulated by resveratrol exposure and directly targets *CDK2*, *CDK4*, *CDK6*, and *BCL-2*, thus promoting cell cycle arrest, diminishing viability, and promoting apoptosis in DOX resistant cells.

Additionally, Citron et al. [40] showed that miR-223 expression levels could predict the effect of CDK4/6 inhibitors and palbociclib (PAB), as well as patients’ prognosis for invasive ductal carcinoma. It was demonstrated that miR-223 was downregulated in luminal and HER2 + BC subtypes. Its low expression was correlated with cell cycle deregulation, poor prognosis, PAB resistance, and low survival in BC patients.

### miRNAs and DNA repair checkpoints

**Fig. 2:**
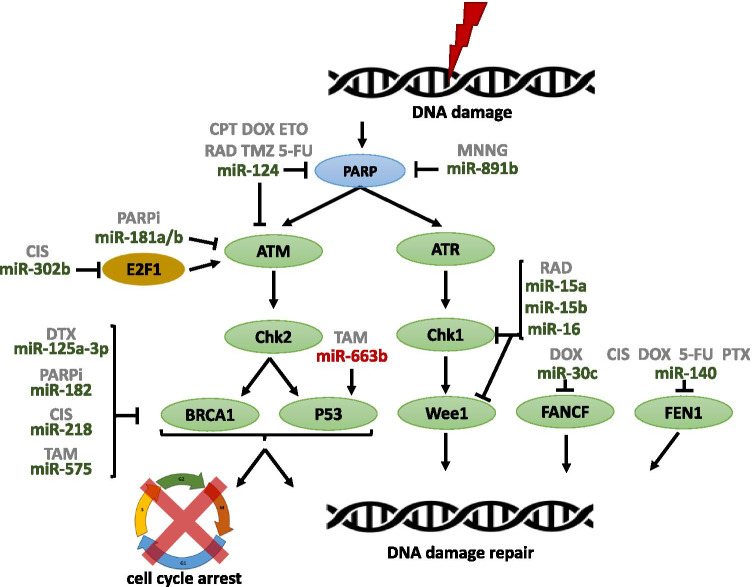
Schematic representation of miRNAs involved in drug resistance through regulating DNA repair checkpoints. Arrows indicate activation and line with a perpendicular line at the end indicates inhibition. miRNAs increasing drug resistance are represented in red color, and miRNAs increasing drug sensitivity are represented in green color. CPT, camptothecin; CIS, cisplatin; DOX, doxorubicin; DTX, docetaxel; ETO, etoposide; MNNG, N-methyl-N′-nitro-N-nitrosoguanidine; PARPi, PARP inhibitors; PTX, paclitaxel; RAD, radiation; TAM, tamoxifen; TRA, trastuzumab; TMZ, temozolomide; 5-FU, 5-fluorouracil

Most chemotherapeutic agents currently in use for cancer therapy induce direct or indirect DNA damage through double-strand breaks (DSB). This kind of DNA damage is repaired by two different mechanisms: homologous recombination (HR) or non-homologous end-joining (NHEJ). HR is an error-free repair and occurs mostly in cells in the S/G2 phase of the cell cycle; Breast Cancer Susceptibility gene 1 (BRCA1), Breast Cancer Susceptibility gene 2 (BRCA2), and RAS associated with diabetes protein 51 (RAD51) are important HR members. NHEJ is an error-prone repair and occurs mostly in the G1 phase of the cell cycle [41]. Ataxia-telangiectasia mutated (ATM), ataxia-telangiectasia and Rad3-related (ATR), and DNA-dependent protein kinase catalytic subunit (DNA-PKCs) are directly activated in response to DNA damage [42]. In this scenario, some miRNAs are involved in controlling the activation of DNA repair pathways (Table 2 and Fig. 2).

**Table.2:** MiRNAs and DNA repair checkpoints (bold, direct targets; italicized, indirectly downregulated targets; italicized and *, indirectly upregulated targets)

MiRNAs	Drug	Targets	Reference
miR-15a miR-15b miR-16	Radiation	**CHK1** **WEE1**	[50]
miR-30c	Doxorubicin	**FANCF** **REV1**	[52]
miR-124	Camptothecin Doxorubicin Etoposide Ionizing radiation Temozolomide 5-fluorouracil	**ATMIN** **PARP1**	[47]
miR-125a-3p	Docetaxel	**BRCA1**	[46]
miR-140	5-fluorouracil Cisplatin Doxorubicin Paclitaxel	**FEN1**	[51]
miR-181a/b	Olaparib	**ATM**	[48]
miR-182	PARP inhibitors: 4-Amino-1, 8- Naphthalimide ABT-888 Olaparib	**BRCA1**	[44]
miR-218	Cisplatin	**BRCA1**	[45]
miR-302b	Cisplatin	**E2F1** *ATM*	[23]
miR-891b	N-methyl-N′-nitro-N-nitrosoguanidine	**PARP1**	[49]

BRCA1 is involved in several cellular pathways that maintain genomic stability, including DNA damage repair, DNA damage-induced cell cycle checkpoint activation, protein ubiquitination, chromatin remodeling, and transcriptional regulation and apoptosis [43]. Therefore, its regulation by miRNAs affects drugs sensitivity. One example is miR-182, which downregulates the expression of *BRCA1*. Moreover, overexpression of this miRNA in BC cells was demonstrated to increase the sensitivity to poly-ADP-ribose-polymerase1 (PARP1) inhibitors (PARPi). Conversely, inhibition of miR-182 enhances BRCA1 levels and induces resistance to PARPi [44]. Besides, the miR-218 [45] and miR-125a-3p [46] have been described to be downregulated in CIS-resistant and DTX-resistant cell lines, respectively. Their ectopic expression in BC drug-resistant cells induced sensitivity through directly targeting *BRCA1* expression. Along with that, miR-124 [47] act as tumor suppressor by sensitizing the BC cells to camptothecin (CPT), etoposide (ETO), DOX, ionizing radiation (RAD), temozolomide (TMZ), and 5-FU via targeting DNA repair-related genes ATM Interactor (*ATMIN*), *PARP1*, and *ATM*. Besides, miR-181a/b act as tumor suppressor by sensitizing the BC cells to PARPi via targeting *ATM* [48]*.* Moreover, *E2F1* has been identified as a direct target gene of miR-302b. E2F1 is not only a master regulator of the G1/S transition but also a positive regulator of ATM. When miR-302b is overexpressed, DNA repair after CIS treatment is ineffective due to a lack of ATM, resulting in induced apoptosis and increased drug effect [23]. Furthermore, miR-891b increases the sensitivity of the BC cells to the cytotoxic effects of the chemotherapeutic DNA-damaging agent N-methyl-N′-nitro-N-nitrosoguanidine (MNNG) by suppressing the expression of PARP1 [49].

Besides, the upregulation of the miR-15 family (miR-15a/15b/16) increases the sensitivity to RAD. This effect is mediated by the downregulation of checkpoint kinase 1 (*CHK1*) and *WEE1*, which participate in RAD-induced G2 arrest and increase cell proliferation [50].

DOX resistance is a major challenge for the treatment of BC. In this context, miR-140 has been described to play tumor suppressor functions through downregulation of Flap Endonuclease 1 *(FEN1*), which is involved in DNA repair and cancer progression. Its role in chemotherapy response has been established since its overexpression sensitized cells to 5-FU, CIS, DOX, and PTX. In addition, it has been demonstrated that the transcription factor Ying Yang 1 (YY1), which promotes miR-140 expression, is downregulated in DOX-resistant models [51]. On the other hand, the role of miR-30c has been linked to DOX response in p53-mutated BC, a well-described feature in chemotherapy resistance. It has been shown that p53 activates miR-30c, which targets the DNA repair protein Fanconi anemia complementation group F protein (FANCF) and the DNA polymerase REV1 (REV1) protein, thus sensitizing cells to DOX. In parallel, reduced miR-30c levels were correlated with p53-mutated BC and associated with lower survival [52].

### miRNAs and cell death

**Fig. 3:**
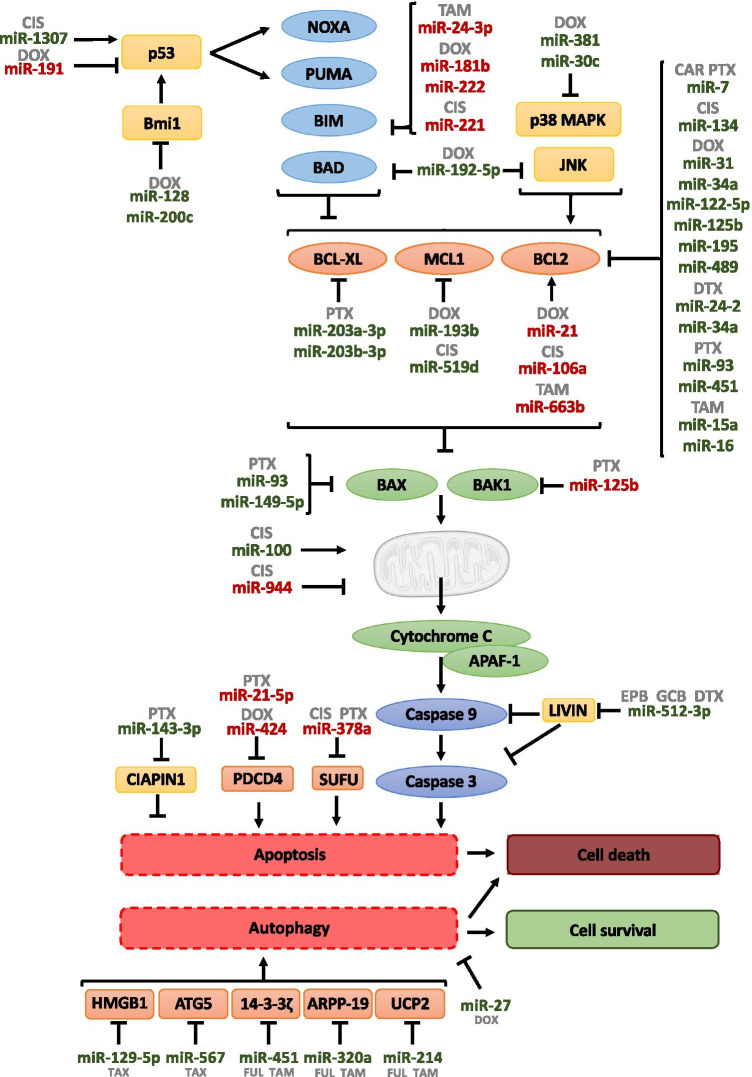
Schematic representation of miRNAs involved in drug resistance through regulating cell death. Arrows indicate activation and line with a perpendicular line at the end indicates inhibition. miRNAs increasing drug resistance are represented in red color, and miRNAs increasing drug sensitivity are represented in green color. CAR, carboplatin; CIS, cisplatin; DOX, doxorubicin; DTX, docetaxel; EPB, epirubicin; GCB, gemcitabine; PTX, paclitaxel; TAM, tamoxifen

Cell death has a prominent role in various physiological and pathophysiological processes in the human body. Apoptosis, autophagy, and programmed necrosis are the three main forms of programmed cell death [53–63]. Due to the fact that miRNAs can modulate cell death, there is an increasing interest in drug-miRNA combination anticancer therapies (Table 3 and Fig. 3).

**Table.3:** MiRNAs and cell death (bold, direct targets; italicized, indirectly downregulated targets; italicized and *, indirectly upregulated targets)

MiRNAs	Drug	Targets	Reference
miR-7	Paclitaxel Carboplatin	**BCL-2**	[76]
miR-15a/16	Tamoxifen	**BCL-2**	[67]
miR-21	Doxorubicin Paclitaxel	**PPIA** BCL-2* **PDCD4**	[69] [97]
miR-24–2	Docetaxel	**BCL-2**	[78]
miR-24-3p	Tamoxifen	**BIM**	[85]
miR-27a	Doxorubicin	**CTH** **SLC7A11** **NFE2L2**	[104]
miR-30c	Doxorubicin	**YWHAZ**	[84]
miR-31	Doxorubicin	**BCL-2**	[70]
miR-34a	Docetaxel Doxorubicin	**BCL-2**	[21] [71]
miR-93	Paclitaxel	*BCL-2* BAX*	[22]
miR-100	Cisplatin	**HAX-1**	[94]
miR-106a	Cisplatin	**BCL-2**	[77]
miR‐122‐5p	Doxorubicin	**BCL-2**	[39]
miR-125b	Doxorubicin Paclitaxel	**BCL-2** **BAK1**	[72] [65]
miR-128	Doxorubicin	**BMI-1**	[93]
miR-129-5p	Taxol	**HMGB1**	[106]
miR-134	Cisplatin	**STAT5B** *HSP90* *BCL-2*	[68]
miR-143-3p	Paclitaxel	**CIAPIN1**	[98]
miR-149-5p	Paclitaxel	BAX*	[66]
miR-181b	Doxorubicin	**BIM**	[88]
miR-191	Doxorubicin	**SOX4**	[91]
miR-192-5p	Doxorubicin	**PPIA** *JNK* *BAD* *CAS9*	[89]
miR-193b	Doxorubicin	**MCL-1**	[79]
miR-195	Doxorubicin	**RAF-1** BCL-2*	[74]
miR-200c	Doxorubicin	**BMI-1** **TRKB**	[92]
miR-203a-3p miR-203b-3p	Paclitaxel	**BCL-XL**	[81]
miR-214	Tamoxifen	**UCP2**	[101]
miR-221	Cisplatin	**BIM** *BAX* *BAK*	[86]
miR-222	Doxorubicin	**BIM**	[87]
miR-320a	Tamoxifen	**ARPP-19** **ERRγ**	[103]
miR-378a-5p	Cisplatin Paclitaxel	**SUFU**	[100]
miR-381	Doxorubicin	**FYN** *pERK* *p38*	[83]
miR-424	Doxorubicin	**PDCD4**	[96]
miR-451	Paclitaxel	**BCL-2**	[75]
miR-451a	Tamoxifen	**14–3-3ζ**	[102]
miR-489	Doxorubicin	**BCL-2**	[73]
miR-512-3p	Epirubicin Gemcitabine Docetaxel	**LIVIN** *CAS3* *CAS9*	[99]
miR-519d	Cisplatin	**MCL-1**	[80]
miR-567	Trastuzumab	**ATG5**	[105]
miR-663b	Tamoxifen	**TP73** BAX*	[35]
miR-944	Cisplatin	**BNIP3**	[95]
miR-1307	Cisplatin	**MDM44**	[90]

The intrinsic or mitochondrial pathway is the most well-studied apoptosis mechanism. Herein, BAX and/or BCL-2 Antagonist/killer 1 (BAK) trigger the release of cytochrome-c from mitochondria to the cytoplasm, where it binds Apoptotic peptidase activating factor 1 (APAF-1) and induces activation of caspases. Activation of BAX/BAK is inhibited by the anti-apoptotic BCL-2 family members, which are bind by pro-apoptotic BH3-only proteins [64]. Therefore, miRNAs that regulate those molecules can increase or decrease the efficacy of anti-cancer drugs. Some examples are miR-125b, which confers resistance to PTX by suppressing the expression of *BAK1* [65], miR-149-5p that was found downregulated in PTX-resistant cells and its overexpression demonstrated to increase BAX expression [66], or miR-663b that confers TAM resistance by indirectly upregulating BAX [35].

Additional miRNAs modulate drug response through regulating the expression of BCL-2 family members. Cittelly et al. found that ER + breast tumors expressing HER2∆16 (an oncogenic isoform of HER2) were resistant to TAM in part through upregulation of the *BCL-2* expression, which is negatively regulated by miR-15a and miR-16 [67]. miR-134 overexpression inhibits signal transducer and activator of transcription 5-B (*STAT5B*), which in turn decreases heat shock protein 90 (HSP90) and BCL-2 levels, resulting in a decreased cell proliferation and increased CIS-induced apoptosis [68]. Regarding DOX, miR-21 [69] acts as an oncomiR that positively regulates *BCL-2* expression leading to drug resistance. On the other hand, several miRNAs, such as miR-31 [70], miR-34a [21, 71], miR‐122‐5p [39], miR-125b [72], and miR-489 [73] have been described as effectors of DOX therapy through direct translational repression of *BCL-2*. Besides, miR-195 was found downregulated in BC cells and tumor samples from multidrug-resistant (MDR) patients. Its upregulation increases the sensitivity to DOX and inhibits rapidly accelerated fibrosarcoma-1 (*RAF-1*), which is a target gene that activates the expression of *BCL-2* and P-glycoprotein (*P-gp*) in BC cells [74]. miR-451 also inhibits the expression of *BCL-2*, which increases PTX-induced apoptosis in BC cell lines [75]. Besides, miR-7 overexpression associates with a better response in BC patients treated with PTX and carboplatin (CAR) chemotherapy due to negatively regulation of *BCL-2* and multidrug resistance-associated protein 1 (*MRP1*) gene [76]. miR-93 is also an enhancer of PTX sensitivity through indirectly inducing BCL-2 inhibition and BAX enhancement [22]. In contrast, miR-106a is upregulated in BC tissue compared to its adjacent tissue, and *in vitro* models showed that the downregulation of this miRNA reduced *BCL-2* and ATP-binding cassette super-family G member 2 (*ABCG2*), enhancing CIS sensitivity [77]. Besides, miR-24-2 increases sensitivity to DTX through targeting *BCL-2*, thus improving the treatment strategy by reducing the side effects of the drugs and minimizing the chemotherapeutic dose [78].

Furthermore, miRNAs miR-193b and miR-519d modulate drug resistance through targeting the BCL-2 family member myeloid cell leukemia 1 (*MCL-1*). miR-193b restored sensitivity to DOX [79], and miR-519d increased CIS-induced cell death in BC stem cells (BCSCs) [80]. Moreover, miR-203a-3p and miR-203b-3p have been reported to decrease of the antiapoptotic protein BCL-XL and to be correlated to PTX sensitivity in BC positively regulated by MYC in cell line models of PTX-responsive BC [81]. Several BCL-2 family members are under control of Jun N-terminal Kinase (JNK) and/or p38 MAPK cascades at a transcriptional and/or post-transcriptional level [82] and several miRNAs regulate apoptosis by modulating those pathways. miR-381 is downregulated in DOX-resistant BC models, and its transient overexpression re-sensitizes cells to this chemotherapeutic agent *in vivo* and *in vitro*. It has been proposed that the *FYN* gene could be part of this process, being identified as a direct target of miR-381 and with a prominent role in the MAPK signaling cascade. Thus, it was confirmed that miR-381 overexpression inhibited the phosphorylation level of extracellular regulated kinase (ERK) and p38 by targeting *FYN* [83]. miR-30c is also downregulated in DOX-acquired resistant models, and its restoration re-sensitizes cells through inhibition of the anti-apoptotic gene Tyrosine 3-Monooxygenase/Tryptophan 5-Monooxygenase Activation Protein Zeta (*YWHAZ*), which specifically regulates the p38 MAPK signaling pathway [84].

Besides, some miRNAs regulate BH3-only proteins such as BCL-2 like 11(commonly called BIM) and BCL-2 associated agonist of cell death (BAD), which are inhibitors of BCL-2 [64]. One example is miR-24-3p, whose overexpression in TAM-acquired resistance BC models has been linked to the direct repression of its target gene *BIM* [85]. In contrast, Ye et al. showed that anti-miR-221-induced BIM upregulation, accompanied by BAK and BAX activation, resulting in CIS-induced apoptosis [86]. miR-222 inhibition also enhanced DOX-induced apoptosis by activating the BIM-caspase pathway [87]. miR-181b also acts as a promoter of DOX resistance in tumor cells by targeting the expression of *BIM*, resulting in maintenance of mitochondrial membrane potential and avoiding activation of caspases cascade after DOX treatment [88]. Moreover, downregulation of miR-192-5p has been observed in BC cell lines with DOX resistance. Its overexpression promotes apoptosis and re-sensitizes DOX-resistant cells by directly targeting peptidylprolyl isomerase A (*PPIA*), which promotes the expression of JNK, BAD, and Caspase 9 (CAS9) [89].

P53 also plays an important role in the intrinsic apoptotic pathway by activating the transcription of BH3-only proteins Superoxide-generating NADPH Oxidase heavy chain subunit A (NOXA) and BCL-2 binding component 3 (PUMA/BBC3) [64]. Wang et al. pointed out miR-1307 as an enhancer of CIS chemosensitivity by targeting *MDM4,* a known p53 inhibitor [90]. Besides, miR-191 establishes a regulatory feedback loop with p53 (its negative regulator) and Sex determining Region Y-box transcription factor 4 (*SOX4*) (its target gene, that in turn promotes p53 activity), highlighting the importance of the p53/miR-191/*SOX4* axis in the regulation of cell death and DOX resistance [91]. Moreover, some other miRNAs contribute to drug resistance by regulating polycomb ring finger *BMI1* proto-oncogene, which is involved in stem maintenance and in the regulation of senescence. Therefore, BMI1 functions as a transcriptional repressor of various genes, including *p19Arf*, which results in degradation of p53, leading to anti-apoptotic effects [92]. Reduction of miR-128 leads to overexpression of BMI1 and ATP Binding Cassette Subfamily C Member 5 (ABCC5) [93], while inhibition of miR-200c leads to overexpression of BMI1 and Tropomyosin receptor kinase B (TRKB) [92]. Due to this fact, the inhibition of miR-128 and miR-200c contributes to DOX resistance in BC [92, 93].

Dysregulation of mitochondrial membrane potential induces intrinsic apoptosis. miR-100 was found to be downregulated in BC cell lines with acquired resistance to CIS. Overexpression of miR-100 showed increased sensitivity to CIS, due to modulation of the HCLS1 associated protein X-1(*HAX-1*), which is an inhibitor of mitochondrial apoptosis that maintains mitochondrial membrane potential in cancer cells [94]. miR-944 inhibitors facilitated CIS-induced loss of mitochondrial membrane potential in resistant models, resulting in intrinsic apoptosis via targeting BCL2 interacting protein 3 (*BNIP3*) [95].

Moreover, programmed cell death 4 (*PDCD4*), a tumor suppressor involved in apoptosis, is targeted by miR-424 and miR-21-5p. Adaption to hypoxia by hypoxia-inducible factor 1 (HIF-1α) has been described to induce miR-424, which in turn suppresses the level of PDCD4, a protein involved in DOX-induced apoptosis, leading to chemoresistance [96]. miR-21-5p has been described as an oncogene, and Tao et al. pointed its role in resistance to PTX *in vitro* and *in vivo* through targeting *PDCD4* gene [97].

miR-143-3p decreases resistance to PTX through regulation of the protein cytokine-induced apoptosis inhibitor 1 (*CIAPIN1*) in the TNBC PTX-resistant mouse model [98]. miR-512-3p has also been described as a potential apoptosis enhancer after treatment with epirubicin (EPB), gemcitabine (GCB), and DTX through direct modulation of the *Livin* gene, which is a negative regulator of apoptosis by binding molecules such as Caspase 3 (CAS3) or CAS9 [99]. Besides, the long non-coding RNA Growth Arrest Specific 5 (lncRNA GAS5) increases sensitivity to CIS and PTX in TNBC by binding miR-378a-5p, which targets the pro-apoptotic gene domain suppressor of fused protein (also known as *SUFU*) [100].

Despite the intrinsic apoptotic pathway is the most studied, there are also other mechanisms of cell death, such as autophagy. This is a process to recycle intracellular components that usually promotes cell survival but can also give rise to cell death through self-digestion [64]. In this context, regulation of autophagy by miRNAs can modulate drug response. There are some miRNAs such as miR-214, and miR-451a that increased the sensitivity of BC cells to TAM and FUL through inhibiting autophagy by targeting Uncoupling protein 2 (*UCP2*) [101] and 14–3-3ζ (a key proliferative and antiapoptotic factor in BC) [102], respectively. miR-320a also inhibits autophagy through targeting phosphoprotein regulated by cAMP (*ARPP-19*) and estrogen-related receptor gamma (*ERRγ*) [103]. Furthermore, miR-27a has been identified as a negative regulator of survival and chemoresistance of BCSCs. The gain of miR-27a function sensitized cells to chemotherapeutic treatment by targeting genes involved in reactive oxygen species detoxification and impaired autophagy after DOX treatment [104].

miR-567 expression is significantly low in TRA-resistant cells. Its exosome-packed gain-of-function enhances apoptosis and reduces autophagy, re-sensitizing cells to TRA, both *in vivo* and *in vitro* models, in part by targeting autophagy-related 5 *(ATG5*) gene, strongly associated with cancer initiation [105]. miR-129-5p has been reported to increase sensitivity to taxol in BC models by inhibiting autophagy. This process included direct regulation of high mobility group box 1 (*HMGB1*), a regulator of autophagy [106].

### miRNAs and receptors

#### ErbB receptors

**Fig. 4:**
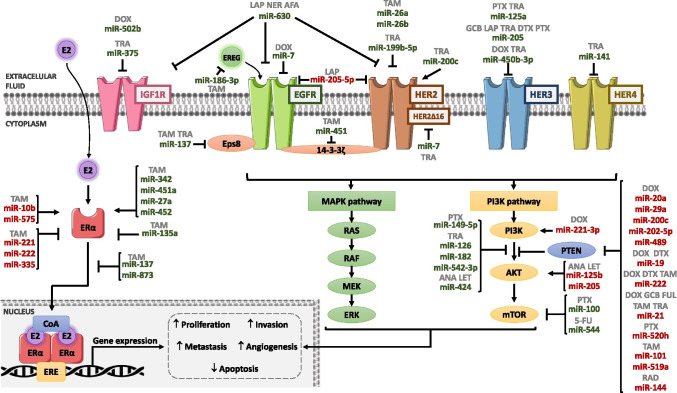
Schematic representation of miRNAs involved in drug resistance through regulating receptors and PI3K/AKT/PTEN/mTOR pathway. Arrows indicate activation and line with a perpendicular line at the end indicates inhibition. miRNAs increasing drug resistance are represented in red color, and miRNAs increasing drug sensitivity are represented in green color. AFA, afatinib; ANA, anastrozole; DOX, doxorubicin; DTX, docetaxel; FUL, fulvestrant; GCB, gemcitabine; LAP, lapatinib; LET, letrozole; NER, neratinib; PTX, paclitaxel; RAD, radiation; TAM, tamoxifen; TRA, trastuzumab; 5-FU, 5-fluorouracil

The ErbB family comprises four transmembrane tyrosine kinase receptors (TKR) that act as receptors for the members of the Epidermal Growth Factor (EGF) family of extracellular protein ligands [107]. Except for HER2 (also known as ErbB2), which has not a described ligand, the EGF Receptor (EGFR) (also known as ErbB1 or HER1), HER3 (also known as ErbB3), and HER4 (also known as ErbB4) form homo- and heterodimers after ligand binding [108]. The ligand-receptor interaction leads to the intracellular TK domain’s autophosphorylation, thus promoting tumor cell survival, proliferation, migration, and invasion by activation of downstream signaling pathways, such as PI3K and MAPK [107]. Dysregulation of receptors, which may occur due to overexpression, amplification, or mutation, is linked to the development of many cancer types, including BC, by promoting its malignant phenotype. [109]. Several miRNAs regulate ErbB receptors enhancing or suppressing its functions, leading to an alteration in therapy response [110, 111] (Table 4 and Fig. 4).

**Table.4:** MiRNAs and receptors (bold, direct targets; italicized, indirectly downregulated targets; italicized and *, indirectly upregulated targets)

MiRNAs	Drug	Targets	Reference
miR-7	Doxorubicin Trastuzumab	**EGFR** **SCR** *HER2Δ16*	[113] [130]
miR-10b	Tamoxifen	**HDAC4** ERα*	[147]
miR-26a miR-26-b	Tamoxifen	**HER2**	[125]
miR-27a	Endoxifen Tamoxifen Toremifene	**ZBTB10-Sp1** ERα*	[154] [155]
miR-125a + miR-205	Paclitaxel Trastuzumab	**HER3** *AKT* *SRC*	[137]
miR-135a	Tamoxifen	**ESR1** **ESRRA** **NCOA1** **PIM2** **MRAS** **LCP1**	[158]
miR-137	Trastuzumab Tamoxifen	**Eps8** SRC3 *GREB1* *TFF1*	[115] [159] [160]
miR-141	Trastuzumab	**HER4**	[141]
miR-182	Trastuzumab	**FOXO1** **Numb** NICD* HES1* HIF-1α* p-AKT*	[127]
miR-186p	Tamoxifen	**EREG**	[116]
miR-199b	Trastuzumab	**HER2**	[126]
miR‐200c	Trastuzumab	**ZEB1** **ZNF217** *HER2*	[128]
miR-205	Trastuzumab Gefitinib Lapatinib Docetaxel	**HER3** **VEGF-A**	[134] [135] [136]
miR-205 (in BCSC)	Lapatinib	**HER2** **p63** *EGFR*	[138]
miR-221 miR-222	Tamoxifen	**ERα** **p27**	[36] [150] [151]
miR-335	Tamoxifen	**ERα**	[152]
miR-342	Tamoxifen	ERα*	[153]
miR-375	Trastuzumab	**IGF1R**	[118]
miR-450b-3p	Doxorubicin Trastuzumab	**HER3**	[140]
miR-451	Tamoxifen	**14–3-3ζ** *EGFR* *HER2*	[122]
miR-451a	Tamoxifen	**14–3-3ζ** ERα*	[102]
miR-452	Tamoxifen	**UGT1A1** ERα*	[156]
miR-502b	Doxorubicin	**IGF1R**	[119]
miR-575	Tamoxifen	**CDKN1B** **BRCA1** ERα*	[149]
miR-630	Afatinib Lapatinib Neratinib	**IGF1R** *EGFR* *HER2*	[117]
miR-873	Tamoxifen	**CDK3** *ERα*	[161] [162]

In BC, EGFR overexpression is associated with larger tumor size and poor clinical outcomes [112]. In this context, several miRNAs have been shown to regulate EGFR and its downstream pathway. One example is miR-7, which is downregulated in BC cells resistant to DOX. The miR-7 overexpression re-sensitized these cells by inhibiting EGFR/PI3K signaling pathway [113]. miR-137 also inhibits EGFR/PI3K pathway by targeting EGFR pathway substrate (*EPS8*), thus increasing TAM and TRA response [114, 115]. Additionally, epiregulin (*EREG*), an agonist of EGFR, was upregulated in TAM-resistant BC cells and negatively regulated by miR-186-3p [116].

Moreover, the miR-630 acts as a tumor suppressor in HER2 overexpressing tumors. The miR-630 overexpression induced a decrease in the protein levels of insulin-like growth factor 1 receptor (IGF1R), EGFR, and HER2, as well as its phosphorylated forms, turning the cells more sensitive to HER-targeting agents (LAP, neratinib (NER), and afatinib (AFA)) [117]. Additionally, *IGF1R* is also targeted by miR-375, which is downregulated in TRA-resistant HER2 + BC cells and patients. The TRA sensitivity is restored by miR-375 overexpression, which reduces IGF1R and p-AKT protein levels [118]. Moreover, Zhang et al. [119] found that miR-502b was upregulated in patients with good response to DOX. They identified that miR-502b directly targets *IGF1R*, and the expression of this miRNA was related to apoptosis induced by DOX through the inactivation of the PI3K pathway.

In ER + BC cells, TAM treatment led to an upregulation of the 14–3-3ζ, a key factor that binds and stabilizes proteins like EGFR or HER2 [120]. Moreover, high levels of 14–3-3ζ were associated with a poor clinical outcome of BC patients treated with TAM [121]. Bergamaschi et al. reported that miR-451 upregulation reduced the expression of 14–3-3ζ and, consequently, restored TAM effectiveness in endocrine therapy-resistant cells, and reduced HER2, EGFR, and MAPK signaling pathway [122].

The HER2 overexpression or amplification is present in approximately 20 to 30% of BCs and is associated with poor prognosis and disease progression once it increases proliferation and metastasis rates [123]. This receptor is the most well-established therapeutic target in HER2 + BC, with several targeted therapies approved, such as TRA or LAP [124]. In ER + BC cells resistant to TAM, an upregulation of HER2 was found. The miR-26a/b inhibits the translation of HER2 and revert TAM resistance [125]. Moreover, miR-199b-5p directly inhibits HER2 expression leading to inhibition of its downstream signaling and its combination with TRA was demonstrated to enhance the treatment efficacy [126]. Additionally, in HER2 + BC cells, miR-182 plays an essential role in the induction of TRA resistance; in turn, TRA treatment reduces miR-182 levels. This miRNA is responsible for the inactivation of Forkhead box protein O1 (FOXO1) and NUMB endocytic adaptor protein (NUMB), whereas it induces Notch Intracellular Domain (NICD), Hes family BHLH transcription factor 1 (Hes1), Hypoxia-inducible factor 1-alpha (HIF-1α), and p-AKT levels. The inhibition of miR-182 alone and/or in combination with TRA reduced the p-AKT levels leading to an upregulation of FOXO1 in TRA-sensitive and TRA-resistant cells, which induced sensitization to TRA [127]. The miR‐200c is another miRNA that regulates HER2 expression in TRA‐resistant cells, and low miR‐200c expression may underlie therapy resistance. TRA sensitivity was restored by miR-200c overexpression, which targets Zinc finger protein 217 (ZNF217) and Zinc finger E-box binding homeobox 1 (ZEB1) [128].

Besides, the HER2Δ16 oncogenic isoform of the HER2 receptor is present in approximately 50% of HER2 + BC and promotes TRA resistance [129]. The re-expression of miR-7 reverted the HER2Δ16 expression induced by TRA through the inactivation of Steroid Receptor Coactivator (SRC) [130].

Moreover, the heterodimerization of HER3 with HER2 plays a vital role in HER2 signaling pathway activation. The HER3 overexpression is related to a higher relapse rate, being a cause of resistance to HER2-mediated therapies [131, 132]. miR-205 functions as tumor suppressor miRNA and improves the response to the TK inhibitors gefitinib (GEF) and LAP and to TRA through abrogating the expression of HER3 along with vascular endothelial growth factor A (VEGF-A) and targets AKT-mediated pathway in BC cells [133, 134]. Moreover, higher miR-205 expression levels were associated with better outcomes in HER2 + BC patients treated with adjuvant TRA [135]. Similar results were obtained with the overexpression of miR-205 in BC cells that increased the sensitivity to DTX. Likewise, the *in vivo* results demonstrated a synergistic effect between miR-205 and DTX [136]. In HER2-overexpressing BC cells, miR-125a and miR-205 were related with HER3 regulation. The combination of these two miRNAs inhibited HER3 expression and reduced the levels of phosphorylated HER3, AKT, and SRC and, as a consequence, the therapeutic efficacy of TRA and PTX against HER2 + BC was enhanced [137]. In contrast, miR-205-5p upregulation was found in BCSCs. In these cells, the high miR-205-5p targeted ERBB pathway and led to LAP resistance. Specifically, miR-205-5p directly repressed HER2 and indirectly EGFR (via miR-205/p63/EGFR regulation) [138]. Additionally, miR-205-5p expression sustained the BC cells stem-like phenotype contributing to tumor aggressiveness and therapy resistance [138, 139]. The miR-450b-3p is another miRNA that directly inhibits HER3 expression and represses the downstream signaling pathway. The overexpression of this miRNA enhanced sensitivity to TRA and DOX by repressing proliferative signal pathways via HER3/HER2/PI3K/AKT axis. Also, the combination of low levels of miR-450b-3p with high expression of HER3 was associated with lower overall survival in BC patients, suggesting a tumor repressor role of miR-450b-3p [140].

Despite the lack of certainty about the HER4 role in BC, miR-141 was proposed as an anti-tumor miRNA. It targets HER4 and is downregulated in the TRA-acquired resistance BC tumor model, where its overexpression enhanced the treatment response [141].

#### Estrogen receptor

ERα is overexpressed in approximately 75% of BCs [142]. It participates in several cellular pathways that regulate gene expression, cell growth, and survival [143]. For ER + BC patients, selective ER modulators (SERMs) such as TAM, selective ER downregulators (SERDs), and blockers of estrogen biosynthesis (aromatase inhibitors) are the main treatment strategies [144]. ER expression is key for estrogen-dependent growth, and its levels are associated with therapy response and prognosis [145]. The acquisition of resistance to endocrine therapies can be related to loss or reduction of ER expression, or loss of estrogen dependence by activation of alternative signaling pathways, among other mechanisms [142]. Several miRNAs have been described to be involved in TAM resistance in BC patients, either through ER modulation or by targeting genes from the ER signaling pathway [146] (Table 4 and Fig. 4).

Five miRNAs were described to be implicated in TAM resistance acquisition. miR-10b and miR-575 increase resistance by indirectly promoting ER. Oppositely, miR-221, miR-222, and miR-335 increase resistance by direct repression of ERα.

miR-10b was found to be overexpressed in TAM-resistant cells, and its expression was inversely correlated with TAM sensitivity. This miRNA directly targets (*HDAC4*) and, consequently, induces TAM resistance [147]. HDAC4 was described as a transcriptional suppressor of ERα expression, thus establishing a possible explanation for the mechanism beyond TAM resistance [148]. In ER + BC cells with acquired TAM resistance, miR-575 was also found to be upregulated. Likewise, miR-575 overexpression was associated with poor outcomes in ER + BC patients. Furthermore, miR-575 targets Cyclin-dependent Kinase Inhibitor 1B (*CDKN1B*) and BRCA1, two proteins that antagonize ERα activity by abolishing ERα-CCND1 interactions, thus contributing to TAM resistance [149]. Besides, miR-221 and miR-222 were overexpressed in HER2 + BC tissues and cells and conferred TAM resistance by targeting p27^kip1^ and ERα [36, 150, 151]. Additionally, miR-335 (miR-335-3p and miR-335-5p) overexpression also enhanced resistance to TAM by repression of ERα and potentially through targeting genes involved in the ERα signaling pathway [152].

Regarding the miRNAs that restore TAM sensitivity, four of them are described to promote ERα: miR-342, miR-451, miR-27a, and miR-452. By contrast, three miRNAs repress ERα: miR-135a by directly targeting Estrogen Receptor 1 (*ESR1*), and miR-137 and miR-873 by targeting ER pathway. In BC tissues, miR-342 expression was positively correlated with ERα expression. Therefore, miR-342 induction in estrogen-dependent BC cells led to ERα upregulation and sensitized cells to TAM [153]. Moreover, Zhen-Ru et al. demonstrated that miR-451a overexpression improved sensitivity to TAM by decreasing 14–3-3ζ expression and reducing the AKT/mTOR signaling pathway activation while increasing ERα expression [102]. miR-27a showed decreased expression in TAM-resistant cells. High miR-27a levels were associated with increased *in vitro* sensitivity to SERMs such as TAM, endoxifen, and toremifene, and higher overall survival in ER + BC patients that underwent endocrine therapies. Additionally, miR-27a overexpression increased the ERα levels, leading to sensitization for SERM treatments [154]. An indirect regulation of ERα by miR-27a via Zinc finger and BTB domain-containing 10-*Sp1* transcription factor (ZBTB10-Sp1) repression had been suggested [155]. Interestingly, in TNBC, miR-452 can be indirectly related to TAM sensitization. In this specific BC subtype, UDP Glucuronosyltransferase Family 1 member A1 (UGT1A1) was found to be a target gene of miR-452. UGT1A1 induced abnormal glycosylation in ERα and decreased its expression. Restoring the miR-452 expression in TNBC cells, the expression and function of UGT1A1 were reverted [156] leading to a rise in ERα expression that is essential to sensitize the cells to TAM [157].

miR-135a is another miRNA related to TAM resistance in BC. *miR-135A1* locus deletion and reduced miR-135a expression were associated with poor prognosis in ER + BC patients. In contrast to the previously described miRNAs, miR-135a seems to be related with ER gene inhibition, as the TAM-mediated loss of miR-135a increased the expression of its target genes *ESR1*, Estrogen-related Receptor Alpha *(ESRRA*), Nuclear receptor Coactivator 1(*NCOA1*), Pim-2 proto-oncogene, serine/threonine kinase *(PIM2*), Muscle RAS oncogene homolog (*MRAS*), and Lymphocyte cytosolic protein 1 *(LCP1*) and consequently led to activation of the ERK1/2 and AKT pathways. So, the crosstalk between miR-135a, ERα, and ERK1/2/AKT induced resistance to TAM in ER + BC cells [158]. Besides, the miR-137/SRC3 axis seems to contribute to TAM resistance in an ER-signaling-dependent manner. In BC patients treated with TAM therapy, high Steroid receptor coactivator-3 (*SRC3*) levels were associated with lower disease-free survival, indicating resistance to therapy [159]. miR-137 suppresses ER signaling by direct targeting *SRC3* and related to the transcription of ER-target genes, such as Growth-Regulating Estrogen Receptor Binding 1 (*GREB1*) and Trefoil Factor 1 (*TFF1*) [160]. Additionally, the miR-873 downregulation has also been associated with TAM resistance by the CDK3-ERα pathway. Low mir-873 levels were found in TAM-resistant cells together with overexpression CDK3, which is a direct target of miR-873 and responsible for ERα phosphorylation. Induction of miR-873 restored TAM sensitivity by decreasing the ERα transcriptional activity and ERα recruitment on ERE sequences in a CDK3-dependent manner [161, 162].

### miRNAs and PI3K/AKT/PTEN/mTOR pathway

PI3K/AKT/PTEN/mTOR pathway regulates multiple cell functions, such as cell proliferation, cell survival, differentiation, and angiogenesis, in normal conditions [163]. Therefore, modulation of this pathway by miRNAs could be one of the mechanisms involved in BC drug resistance. This pathway is activated after a particular ligand, such as insulin or growth factors, binds to TKs or G-protein-coupled-receptors in the cell membrane. Subsequently, PI3K activation promotes the phosphorylation of phosphatidylinositol 4, 5–biphosphate (PIP2) to phosphatidylinositol 3,4,5–triphosphate (PIP3), which initiates the signaling cascade that implicates AKT phosphorylation and activation. After that, mTOR is activated through AKT and triggers several biological functions, such as cell proliferation, invasion, and angiogenesis [163]. PTEN is a master negative regulator of the pathway by preventing the activation of PIP3. It is widely described in the literature that PTEN expression is disrupted in many types of tumors due to genetic alterations or epigenetic regulations, producing a continuous PI3K activation and initiating epithelial-to-mesenchymal transition (EMT) process and drug resistance [164].

miRNAs that directly target PTEN were found to be dysregulated in BC (Table 5 and Fig. 4). Liang et al. [165] demonstrated that PTEN downregulation in DOX and DTX-resistant BC tumors was controlled by miR-19, which has an oncogenic effect. Besides, numerous authors focused on the study of miR-21, which also modulates *PTEN* expression and regulates sensitivity to different drugs such as DOX [166], TRA [167], TAM [168], FUL [168], and GCB [169]. Moreover, miR-144 overexpression was correlated with increased resistance against RAD in BC cell lines. This miRNA increased proliferation, migration, and invasion by downregulating *PTEN* expression and activating the PI3K pathway [170]. The downregulation of PTEN was also directly linked to miR-202-5p [171], miR-20a [172], and miR-29a [173] and their overexpression increased cell proliferation and DOX resistance, and decreased apoptosis levels through the PI3K/AKT activation pathway. Furthermore, miR-200c was found downregulated in DOX-resistant BC cells. Its direct target *ZEB1* negatively regulates E-cadherin (CDH1) and PTEN, thus activating the PI3K pathway, conferring drug resistance [174]. Interestingly, miR-489 was found to be downregulated in DOX-resistant BC tissues, which was correlated with poor prognosis. Its overexpression increased chemosensitivity and inhibited proliferation, migration, and invasion through targeting Spindlin 1 (*SPIN1*), which activated the PI3K/AKT pathway involved in chemoresistance. Moreover, Vav guanine nucleotide exchange factor 3 (*VAV3*), *BCL-2*, and *AKT3*, which are involved in cell growth, were also found to be direct targets of miR-489 [73]. Zhong et al. [173] and Shen et al. [175] demonstrated that miR-222 promoted resistance to both DOX and DTX by targeting *PTEN* and indirectly inhibiting FOXO1, which is an AKT pathway inhibitor implicated in tumor suppressor functions such as cell cycle, apoptosis regulation, and cell differentiation. Additionally, Gu et al. [176] found that miR-222 promoted resistance to TAM by targeting *PTEN* as a result of the decrease of *GAS5*, which is a molecular sponge for miR-222. Moreover, miR-520 h was described as a PTEN modulator by targeting OTU Deubiquitinase 3 (*OTUD3*), a deubiquitinase that controls PTEN degradation. This miRNA promoted PTX resistance and poor prognosis in BC patients, increasing proliferation and reducing PTX-mediated apoptosis [177]. Likewise, Sachdeva et al. [178] found that estradiol controlled miR-101 functions. In the absence of estradiol, miR-101 indirectly regulated PTEN expression through its direct target Membrane Associated Guanylate kinase, WW and PDZ domain containing 2 (*MAGI-2*), then activating AKT and producing TAM resistance. In addition, miR-519a was identified upregulated in TAM-resistant cells, and *PTEN* was described as its direct target among other genes such as *RB1* and *CDK1A* [33]. It was demonstrated that patients with high levels of miR-519a presented lower survival and worse response to TAM treatment.

**Table.5:** miRNAs and PI3K pathway (bold, direct targets; italicized, indirectly downregulated targets; italicized and *, indirectly upregulated targets)

MiRNAs	Drug	Targets	Reference
miR-19	Doxorubicin Docetaxel	**PTEN**	[165]
miR-20a	Doxorubicin	**PTEN**	[172]
miR-21	Doxorubicin Gemcitabine Fulvestrant Tamoxifen Trastuzumab	**PTEN**	[166] [169] [168] [167]
miR-29a	Doxorubicin	**PTEN**	[173]
miR-100	Paclitaxel	**mTOR**	[180]
miR-101	Tamoxifen	**MAGI-2** *PTEN*	[178]
miR-125b	Anastrozole Letrozole	AKT/mTOR*	[184]
miR-126	Trastuzumab	**PIK3R2**	[182]
miR-144	Radiation	**PTEN**	[170]
miR-149-5p	Paclitaxel	**MYD88**	[66]
miR‐200c	Doxorubicin	**ZEB1** *PTEN*	[174]
miR-202-5p	Doxorubicin	**PTEN**	[171]
miR-205	Anastrozole Letrozole	AKT/mTOR*	[184]
miR-221-3p	Doxorubicin	**PIK3R1**	[183]
miR-222	Doxorubicin Docetaxel	**PTEN** *FOXO1*	[175] [173] [176]
miR-424	Anastrozole Letrozole	*AKT/mTOR*	[184]
miR-489	Doxorubicin	**SPIN1** **VAV3** **AKT3**	[73]
miR-519a	Tamoxifen	**PTEN** **CDKN1A**	[33]
miR-520 h	Paclitaxel	**OTUD3** *PTEN*	[177]
miR-542-3p	Trastuzumab	***PI3K pathway***	[181]
miR-544	5-Fluorouracil	**mTOR**	[179]

miR-149-5p was found downregulated in PTX-resistant cells, and Myeloid Differentiation primary response gene 88 (*MYD88*), a PI3K/AKT pathway activator, has been described as its direct target. Besides, higher expression of BAX was observed, which led to enhanced apoptosis [66]. In another way, miR-544 [179] and miR-100 [180] directly target *mTOR* and have been associated with 5-FU and PTX sensitivity, respectively, in BC cell lines and patients.

Furthermore, miR-542-3p expression was induced by TRA treatment in HER2 + BC cell lines. The re-expression of miR-542-3p increased TRA sensitivity and led to apoptosis through blockage of G1/S checkpoint and lower PI3K pathway activation [181]. Additionally, in TRA-resistant HER2 + BC cells, Phosphoinositide-3-Kinase Regulatory subunit 2 (*PIK3R2*) was described as a direct target of miR-126. Low miR-126 levels were translated into *PIK3R2* upregulation leading to enhanced drug resistance and higher migration and invasion rates [182]. Moreover, miR-221-3p promoted resistance to DOX in BC by decreasing *PIK3R1* expression and consequent PI3K pathway activation [183]. Besides modulation of AKT/mTOR pathway has also been involved in aromatase inhibitors resistance, as shown by Vilquin et al. that found that overexpression of miR-125b and miR-205, and downregulation of miR-424 confer resistance to letrozole (LET) and anastrozole by activating the AKT/mTOR pathway [184].

### miRNAs and stemness and epithelial to mesenchymal transition

Nowadays, it has been demonstrated that BC comprises a heterogeneous population of cells. Those can be roughly classified into two main populations: BCSCs and differentiated cells. BCSCs are a minor population of cells that carry a high tumorigenic capacity and are involved in resistance to different therapies [185–192].

Several molecular pathways are involved in BCSCs-phenotype regulation; among them, the most important is EMT. This process occurs during cancer development, where there is a loss of expression of epithelial-associated molecules like CDH1 and an increase of mesenchymal-associated molecules such as N-cadherin (CDH2), vimentin (VIM), and fibronectin (FN1) [188, 193]. As a result, the cells increase their invasion and migration capacity [185, 186, 188] and can nest in different tissues where they can proliferate to originate new tumors in a process known as metastasis [188]. There are different molecules involved in the induction of EMT, including Snail Family Transcriptional Repressor 1 (SNAI1), ZEB1/2, Twist Family BHLH Transcription Factor 1/2 (TWIST1/2) [194, 195], and some growth factors such as Transforming Growth Factor-beta (TGF-β), EGF, and Tumor Necrosis Factor-alpha (TNFα) [193].

**Fig. 5:**
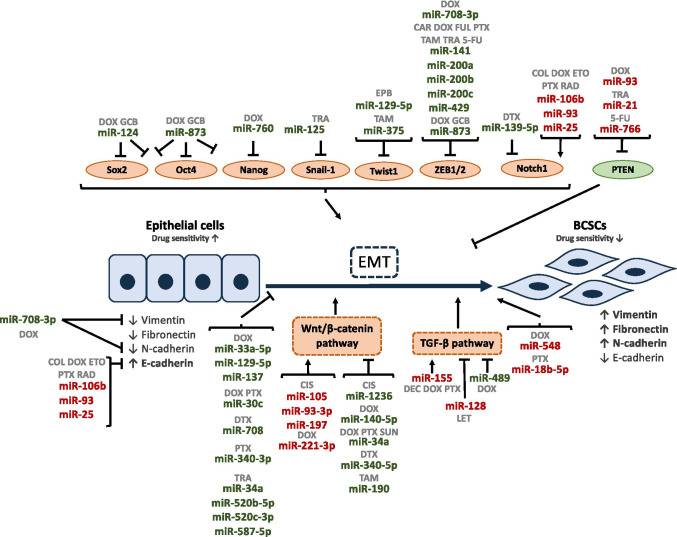
Schematic representation of miRNAs involved in drug resistance through regulating stemness and epithelial to mesenchymal transition. Arrows indicate activation and line with a perpendicular line at the end indicates inhibition. miRNAs increasing drug resistance are represented in red color, and miRNAs increasing drug sensitivity are represented in green color. CAR, carboplatin; CIS, cisplatin; COL, colchicine; DEC, decitabine; DOX, doxorubicin; DTX, docetaxel; EPB, epirubicin; ETO, etoposide; FUL, fulvestrant; GCB, gemcitabine; LET, letrozole; PTX, paclitaxel; RAD, radiation; SUN, sunitinib; TAM, tamoxifen; TRA, trastuzumab; 5-FU, 5-fluorouracil

In this scenario, miRNAs play an important role in regulating stemness and EMT by targeting several genes involved in these two pathways (Table 6 and Fig. 5). miR-200 family is the most studied miRNA family across those involved in EMT regulation. It comprises five members: miR-141, miR-200a, miR-200b, miR-200c, and miR-429 [196], which can downregulate *ZEB1* and *ZEB2* [196–198]. As a result, it has been shown that overexpression of miR-200 reverses EMT in different cancer cell lines [199]. Among the miR-200 family, miR-200c is the most studied member. It is able to reduce migration and invasion in BC by inhibiting EMT [199, 200]. It has been illustrated that miR-200b/c acts as a tumor suppressor by targeting multiple genes involved in EMT and metastasis, thus having a role in drug resistance in BC. This family of miRNAs increases sensitivity to chemotherapeutic drugs, including CAR and DOX [174, 201, 202], PTX [203, 204], 5-FU [205], and to other drugs such as FUL, TAM [206, 207], and TRA [128, 208, 209] through downregulation of EMT inductors. In addition, miR-200c has been demonstrated to be upregulated in BC patients who experienced low response to neoadjuvant chemotherapy compared to those with high response to the treatment [210]. *ZEB1* is also regulated by other miRNAs. One of these miRNAs is miR-708-3p, which acts as a tumor suppressor miRNA, targeting two EMT markers, *CDH2* and *VIM*, resulting in repression of EMT, metastasis, and improvement of sensitivity to DOX *in vitro* and *in vivo* [211]. Wang et al. demonstrated that miR-873 overexpression increases GCB sensitivity while its downregulation promotes drug resistance and increases mesenchymal phenotype by CDH1 downregulation and upregulation of ZEB1 target genes such as Yes1-Associated Transcriptional Regulator (*YAP1*) [212]. Gao et al. showed that miR-873 is also associated with attenuation of DOX resistance through direct targeting Programmed Death-ligand 1 (*PD-L1*). PD-L1 inhibition led to decreased capacity of mammosphere formation *in vitro*, tumor formation *in vivo*, and repression of stemness markers such as Octamer-binding transcription factor 4 (OCT4), Aldehyde dehydrogenase 1 family (ALDH1A1), NANOG, and SRY (sex determining region Y)-box transcription factor 2 (SOX2), thus increasing the efficacy of DOX treatment [213].

**Table.6:** MiRNAs and EMT/CSC (bold, direct targets; italicized, indirectly downregulated targets; italicized and *, indirectly upregulated targets)

MiRNAs	Drug	Targets	Reference
miR-18b-5p	Paclitaxel	**DOCK4**	[244]
miR-21	Trastuzumab	**PTEN**	[249] [167]
miR-25	Doxorubicin γ-radiation Etoposide Colchicine Paclitaxel	**EP300** ***CDH1***	[234] [235]
miR-30c	Doxorubicin Paclitaxel	**TWF1**	[214]
miR-33a-5p	Doxorubicin	**eIF5A2**	[215]
miR-34a	Doxorubicin Paclitaxel Sunitinib Trastuzumab	**NOTCH1** **WNT1** **CD44**	[227] [228] [229] [230] [231]
miR-93	Doxorubicin γ-radiation Etoposide Colchicine Paclitaxel	**EP300** *CDH1* **PTEN**	[234] [235] [247]
miR-93-3p miR-105	Cisplatin	**SPFR1** *Wnt/β-catenin pathway*	[237]
miR-106-b	Doxorubicin γ-radiation Etoposide Colchicine Paclitaxel	**EP300** *CDH1*	[234] [235]
miR-124	Doxorubicin	**STAT3** *ALDH1* *OCT4* *SOX2* *HIF-1*	[232]
miR-125	Trastuzumab	**SNAI1**	[222]
miR-128	Letrozole	**TGF-βRI**	[270]
miR-129-5p	Doxorubicin Epirubicin	**SOX4** **TWIST1**	[216] [217]
miR-137	Doxorubicin	**DUSP4**	[218]
miR-139-5p	Docetaxel	**NOTCH1**	[226]
miR-140-5p	Doxorubicin	**WNT1** *OCT4* *ALDH1*	[240]
miR-141-3p	Trastuzumab	**CDK8** *SMAD2/3* *TGF-β pathway*	[209]
miR-155	Doxorubicin Paclitaxel	**FOXO-3a** **C/EBP-β** BMI1* SLUG* SNAI1* EZH2* *CDH1* *TGF-β* **TSPAN5**	[245] [246]
miR-190	Tamoxifen	**SOX9**	[241]
miR-197	Cisplatin	**NLK**	[239]
miR-200	Paclitaxel	**Jagged2**	[203]
miR-200b	5-fluorouracil Carboplatin Doxorubicin Tamoxifen Fulvestrant	**FN1** **ZEB1** **FN1** **ARRDC3** *CDH1* *VIM* **ZEB1/2** *CDH2* *VIM* *SLUG* **c-MYB**	[201] [205] [206] [207]
miR-200c	Carboplatin Doxorubicin Paclitaxel Fulvestrant Tamoxifen Trastuzumab	**ZEB1/2** CDH1* *PTEN* *Akt pathway* *VIM* **SOX2** *CDH2* *SLUG* *VIM* **c-MYB** **ZNF217** *TGF-β pathway* **Jagged2** **BMI1** *NOTCH* *WNT* *Hedgehog pathway*	[174] [202] [204] [206] [207] [128] [208]
miR-221-3p	Doxorubicin	**DKK2**	[238]
miR-340-3p	Paclitaxel	**YWHAZ** *SLUG* *SNAI1* *VIM* CDH1*	[219]
miR-340-5p	Docetaxel	**LGR5** *Wnt/β-catenin pathway*	[220]
miR-375	Tamoxifen	**HOXB3** **MTDH**	[224] [225]
miR-489	Doxorubicin	**SMAD3**	[221]
miR-520b-5p	Trastuzumab	**CD44**	[231]
miR-520c-3p	Trastuzumab	**CD44**	[231]
miR-548	Doxorubicin	**PBLD**	[243]
miR-587-5p	Trastuzumab	**CD44**	[231]
miR-708-3p	Doxorubicin Docetaxel	**ZEB1** **CDH2** **VIM** **CD47**	[211] [233]
miR-760	Doxorubicin	**Nanog** ***VIM*** ***CDH1***	[223]
miR-766	5-fluorouracil	**PTEN** *VIM* *CDH2* *SNAI1*	[248]
miR-873	Gemcitabine Doxorubicin	**ZEB1** CDH1* *YAP1* **PD-L1** *OCT4* *ALDH1A1* *NANOG* *SOX2*	[212] [213]
miR-1236	Cisplatin	**SLC9A1**	[242]

Several miRNAs have been related to DOX sensitivity. Among them, miR-30c increases the sensitivity to DOX and PTX and reverts some EMT traits by inhibiting the expression of the cytoskeleton gene Twinfilin-1 (*TWF1*) [214]. Guan et al. showed miR-33a-5p association with DOX response in TNBC cells. Its overexpression increases DOX sensitivity by decreasing EMT through its direct target, Eukaryotic Translation Initiation Factor 5A2 (*eIF5A2*) [215]. miR-129-5p has also been linked with EMT and drug resistance. Luan et al. found miR-129-5p expression lower in DOX-resistant cells and confirmed that its overexpression reverts EMT through SOX4 modulation and increases sensitivity to DOX [216]. In addition, a high miR-129-5p expression also promotes EPB sensitivity due to EMT inhibition by direct *TWIST1* repression [217]. miR-137 also attenuates DOX resistance by directly targeting Dual Specificity Phosphatase 4 (*DUSP4*) and decreasing EMT *in vitro* and *in vivo* [218]. Yan et al. found miR-340-3p to be downregulated in MCF7-PTX-resistant cells while LncRNA H19, which binds and regulates miR-340, was overexpressed. miR-340-3p mimics transfection inhibited SLUG, SNAI1, and VIM expression; increased CDH1; and restrained migration and invasion properties due to its direct regulation of *YWHAZ* [219]. The counterpart strand of this miRNA, miR-340-5p, inhibited DTX resistance through downregulation of Leucine-rich repeat-containing G-protein coupled receptor 5 (*LGR5*) via suppressing the Wnt/β-catenin pathway [220]. Moreover, Jiang et al. studied the effect of miR-489 in DOX-resistant cells. This miRNA is downregulated in resistant cells, and its overexpression demonstrated to revert chemoresistance through regulation of Smad3, which plays an important role in TGF‐β‐induced EMT [221]. TRA-resistant cells generated by Dong et al. showed miR-125 downregulation and overexpression of its regulator Terminal differentiation‐induced non‐coding RNA (lncRNA TINCR). Upregulation of miR-125 and consequent inhibition of TINCR suppresses EMT and migration ability by directly binding miRNA to *SNAI1*. These results suggest that TINCR/miR-125/SNAI1 would be a promising target to overcome TRA resistance in HER2 + BC [222].

Several miRNAs have been studied due to their role in drug resistance by direct or indirect regulation of stemness. Among them, Hu et al. demonstrated that miR-760 is downregulated in DOX-resistant cell lines and chemoresistant BC tissues. Its higher expression *in vitro* directly targets *NANOG* and regulates *VIM* and *CDH1*, leading to higher growth inhibition by DOX [223]. Moreover, it has been proved that miR-375 overexpression increases sensitivity to TAM mediated by downregulation of Homeobox B3 (*HOXB3*) and metadherin (*MTDH*), which are mainly involved in regulating some features of CSCs, as well as EMT [224, 225].

NOTCH1 is a receptor involved in the maintenance and self-renewal of BCSCs [187, 194]. miR-34a and miR-139-5p have been described to target *NOTCH1* directly. Consequently, their upregulation increases the sensitivity to several drugs and reduces some CSCs traits. miR-139-5p inhibits migration and invasion, induces cell cycle arrest, and increases sensitivity to DTX [226], whereas miR-34a has been described to be downregulated in breast tumors and to increase sensitivity to DOX [227] and PTX [228]. miR-34a has also been described to be involved in the multi-target TKR inhibitor sunitinib response by decreasing the invasion capacity of MCF7 BC cells by directly targeting Wnt Family Member 1 (*WNT1*) [229]. *CD44* is also a target of miR-34a, and it is closely linked to BC progression and particularly to TRA resistance due to its ability to prevent the binding between this antibody and HER2 receptor [230, 231]. miR-34a together with miR-520c-3p, miR-520b-5p, and miR-587-5p inhibit metastasis and cancer stemness in BC by targeting *CD44*. Upregulation of these miRNAs increases the efficiency of HER2-targeting strategies and would be foreseeable that their use may conquer TRA resistance more effectively [231].

Signal Transducer and Activator of Transcription 3 (STAT3) is also upregulated in BCSCs compared to epithelial cancer cells. Liu et al. found upregulation of STAT3 in DOX-resistant cells while downregulation of miR-124. Suppression of *STAT3* by direct miR-124 targeting reduced drug resistance, migration, and expression of ALDH1, OCT4, SOX2, and HIF-1α [232].

Tan et al. found miR-708 to be downregulated in BCSCs compared to adherent cells. Inhibition of miR-708 leads to a higher ability of mammosphere formation *in vitro* and tumor formation *in vivo*, and it associates to better response to chemotherapy and higher survival in patients. *CD47* was validated as a direct target of miR-708, and its inhibition reduced the self-renewal capacity, increased phagocytosis of BCSCs by macrophages, and sensitivity to DTX [233].

Increasing evidence suggests that the function of some other miRNAs is mainly involved in the EMT process that plays a crucial role in MDR by the promotion of tumor metastasis. Zhou et al. demonstrated that the miR-106b∼25 cluster is involved in DOX-resistance [234], and Hu et al. related its overexpression in BC cells to resistance to DOX, γ-RAD, ETO, colchicine, and PTX [235]. miRNAs like miR-106b, miR-93, and miR-25 were demonstrated to activate EMT by inhibition of E1A Binding Protein P300 (EP300), a transcriptional activator of CDH1 [234, 235]. Furthermore, this cluster has also been demonstrated to upregulate NOTCH1 at the post-transcriptional level by targeting the E3 ubiquitin ligase Neural precursor cell expressed developmentally downregulated 4-like (NEDD4L). As a result, it induces BC tumor initiation *in vitro* and *in vivo* [236].

The Wnt/β-catenin signaling pathway is also involved in stemness in BC. This pathway has been demonstrated to be regulated by several miRNAs such as miR-105 and miR-93-3p. Those miRNAs target Secreted Frizzled Related protein 1 (*SPFR1*), which is a suppressor of the Wnt/β-catenin signaling pathway. Due to this fact, Li et al. demonstrated that those miRNAs promote CIS resistance [237]. In the same trend, miR-221-3p is upregulated in DOX-resistant cells and non-responder patients’ tumor samples. miR-221-3p mimics decreased growth inhibition rate of DOX through targeting Dickkopf Wnt signaling pathway inhibitor 2 (*DKK2*), a critical modulator of Wnt/β-catenin signaling pathway, and consequently upregulated ATP Binding Cassette Subfamily B Member 1 (ABCB1). Simultaneously, miR-221 is regulated by lnc-RNA-GAS5. Thus, Chen et al. propose the GAS5-miR-221-DKK2 axis as a potential strategy to beat chemoresistance in BC by inactivation of the Wnt pathway [238].

Moreover, Tang et al. elucidated the role of miR-197 on chemotherapy resistance. The authors observed that Taurine Upregulated 1 (TUG1) could sponge miR-197 and simultaneously miR-197 represses Nemo Like Kinase (*NLK*). Interestingly, NLK is a negative regulator of Wnt signaling, which mediates stemness and chemoresistance. In this context, overexpression of miR-197 is associated with low expression of TUG1 and NLK and consequently Wnt pathway activation, thus decreasing CIS sensitivity [239].

On the other hand, several microRNAs increase sensitivity to drugs by inhibiting the Wnt/β-catenin signaling pathway. In this context, Wu et al. found miR-140-5p markedly downregulated in BCSCs compared to non-BCSCs. Overexpression of miR-140-5p decreased self-renewal ability and sensitized BC cells to DOX through directly targeting *WNT1 in vitro* and *in vivo*. Wnt pathway inhibition decreased stem cell markers such as OCT4 and ALDH1 and pumps such as ATP Binding Cassette Subfamily B Member 1 (ABCB1) [240]. Another example is miR-190, whose overexpression rendered cells high sensitivity to TAM *in vitro* and *in vivo* and decreased mammosphere formation and BCSCs population through directly targeting SRY (Sex determining Region Y)-box transcription 9 (*SOX9*) and consequently inactivating the Wnt/β-catenin pathway [241]. Moreover, Jia et al. transferred adipose mesenchymal stem cell-derived exosomes to CIS-resistant BC cells and increased drug sensitivity. It was identified miR-1236 as a specific cargo of exosomes, which targets explicitly Solute Carrier Family 9 Member A1 (SLC9A1), a protein overexpressed in resistant cells with a role in migration capacity by activating the Wnt/β-catenin axis [242].

Liang et al. found miR-548 overexpressed in the MDA-MB-231 DOX-resistant cell line. miR-548, which is regulated by circular RNA CircKDM4C, promotes EMT, invasion ability, and DOX resistance; thus, it has been proposed as an oncogenic miRNA. Phenazine Biosynthesis Like Protein Domain Containing (*PBLD*) was validated as a miR-548 target and is involved in response to chemotherapy and EMT [243].

Wang et al. identified miR-18b-5p to be upregulated in PTX-resistant BC cells. LncRNA AC073284.4 is downregulated in resistant cells and directly regulates miR-18b-5p by a negative correlation, while miR-18b-5p targets Dedicator of Cytokinesis 4 (*DOCK4*), which has a role in adhesion, invasion, and metastasis capacity [244].

Carvalho Santos et al. and Wu et al. found miR-155 upregulation in BCSCs and drug-resistant cells [245, 246]. Indeed, overexpression of miR-155 increased the population of stem-like cells. Exosomes secreted from chemoresistant cells were miR-155 enriched, and their transfer to chemosensitive cells was able to decrease the effect of DOX and PTX and increase their migration potential by upregulating EMT markers expression such as BMI1, SLUG, SNAI1, and Enhancer Of Zeste 2 Polycomb Repressive Complex 2 Subunit (EZH2) and decrease of CDH1 through direct inhibition of Forkhead box O3a (*FOXO-3a*) and CCAAT-enhancer-binding protein (*C/EBP-β*) which causes loss of TGF-β inhibitory effect [245]. Tetraspanin 5 (*TSPAN5*) is also a direct target of miR-155, which reduces stemness and decitabine resistance in TNBC cells [246].

Moreover, PTEN has also been described to be involved in EMT inhibition by several authors. Due to this fact, miRNAs targeting PTEN can be potential EMT inductors, giving rise to drug resistance. Among them, miR-93, miR-766, and miR-21 have been validated as a direct regulators of PTEN. miR-93 was overexpressed in DOX-resistant cells, and it decreased sensitivity to DOX by inducing EMT [247]. In the same trend, miR-766 increased invasion and migration capacity and expression of VIM, CDH2, and SNAI1 and promoted 5-FU resistance [248]. Besides, miR-21 has also been demonstrated to involve the induction of EMT and resistance to TRA in HER2 + BC [167, 249].

### Efflux pumps and miRNAs

**Fig. 6:**
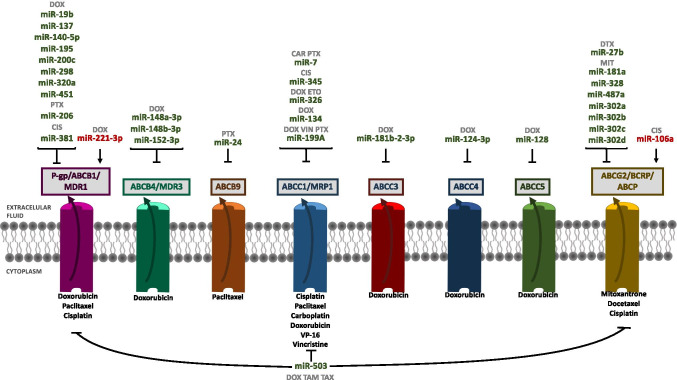
Schematic representation of miRNAs involved in drug resistance through regulating efflux pumps. Arrows indicate activation and line with a perpendicular line at the end indicates inhibition. miRNAs increasing drug resistance are represented in red color, and miRNAs increasing drug sensitivity are represented in green color. CAR, carboplatin; CIS, cisplatin; DOX, doxorubicin; DTX, docetaxel; ETO, etoposide; MIT, mitoxantrone; PTX, paclitaxel; TAM, tamoxifen; TAX, taxol; VIN, vincristine

Another mechanism of resistance to chemotherapy is mediated by ATP-dependent efflux pumps, which decrease the intracellular concentration of drugs. These proteins belong to the family of ATP-binding cassette (ABC) transporters, which comprises 48 members classified in seven subfamilies (ABCA-ABCG) [250]. Due to this fact, some authors have studied the regulation of different ABC transporters by miRNAs in cells with acquired resistance to chemotherapy (Table 7 and Fig. 6).

**Table.7:** MiRNAs and pumps (bold, direct targets; italicized, indirectly downregulated targets)

MiRNAs	Drug	Targets	Reference
miR-7	Cisplatin Carboplatin Paclitaxel	**ABCC1**	[262] [76]
miR-19b	Doxorubicin	**ABCB1**	[254]
miR-24	Paclitaxel	**ABCB9**	[261]
miR-27b	Docetaxel	**ENPP1** *ABCG2*	[275]
miR-106a	Cisplatin	*ABCG2*	[77]
miR-124-3p	Doxorubicin	**ABCC4**	[268]
miR-128	Doxorubicin	**ABCC5**	[93]
miR-134	Doxorubicin	**ABCC1**	[264]
miR-137	Doxorubicin	**ABCB1**	[251]
miR-140-5p	Doxorubicin	***ABCB1***	[240]
miR-148a-3p miR-148b-3p miR-152-3p	Doxorubicin	**SPIN1** *ABCB4* *CYP2C8* *UGT2B4* *UGT2B17*	[258]
miR-181a	Mitoxantrone	**ABCG2**	[271]
miR-181b-2-3p	Doxorubicin	**ABCC3**	[266]
miR-195	Doxorubicin	**RAF-1** *ABCB1*	[74]
miR-199a	Doxorubicin Vincristine Paclitaxel	**ABCC1**	[265]
miR-200c	Doxorubicin	**ABCB1**	[210]
miR-206	Paclitaxel	**ABCB1**	[256]
miR-221-3p	Doxorubicin	**DKK2** *ABCB1*	[238]
miR-298	Doxorubicin	**ABCB1**	[252]
miR-302a/b/c/d	Mitoxantrone	**ABCG2**	[274]
miR-320a	Doxorubicin	**TRPC5** *NFAT3* *ABCB1*	[255]
miR-326	Doxorubicin VP-16	**ABCC1**	[263]
miR-328	Mitoxantrone	**ABCG2**	[272]
miR-345	Cisplatin	**ABCC1**	[262]
miR-381	Cisplatin	**ABCB1**	[257]
miR-451	Doxorubicin	**ABCB1**	[253]
miR-487	Mitoxantrone	**ABCG2**	[273]
miR-503	Doxorubicin Tamoxifen Taxol	**eIF4G** *ABCB1* *ABCC1* *ABCG2*	[276]

The most studied ABC transporter is the ABCB1, also called P-gp or MDR1, which is able to transport hydrophobic drugs with a neutral or positive charge like DOX [250]. Zhu et al. Chen et al., Bao et al. and Kovalchuk et al. demonstrated that miR-137, miR-200c, miR-298, and miR-451 were downregulated in DOX-resistant cells, respectively. The overexpression of those miRNAs increased sensitivity to DOX by targeting *ABCB1* [210, 251–253]. miR-195 also enhances DOX sensitivity through indirect inhibition of *ABCB1*, which is mediated by direct targeting of *RAF-1* [74]. Moreover, Thorne et al. demonstrated that overexpression of miR-19b sensitizes cells to DOX via non-canonical binding to *ABCB1* with the RNA-binding protein HuR [254]. The miR-320a was also reported to be an indirect inhibitor of *ABCB1* downregulated in DOX-resistant cells. He et al. demonstrated that miR-320a targets Transient Receptor Potential Channel C5 *(TRPC5*), which induces the activation of Nuclear Factor of Activated T-cells isoform C3 (NFATC3) and stimulates *ABCB1* expression. It was also demonstrated that the transcription factor v-ets erythroblastosis virus E26 oncogene homolog 1 (ETS-1) is an inhibitor of miR-320a and it is upregulated in chemoresistant cells. These results were validated in samples from chemoresistant patients [255]. In this line, miR-221-3p is another inductor of ABCB1 expression through directly targeting Dickkopf WNT Signaling Pathway Inhibitor 2 (*DKK2*) [238]. As previously mentioned, miR-140-5p also promoted sensitivity to DOX through downregulation of *ABCB1*, maybe by targeting the Wnt-1/β-catenin signaling pathway [240]. ABCB1 is not only able to transport DOX but also PTX and CIS. In line with this, Wang et al. demonstrated that lncRNA ferritin heavy chain 1 pseudogene 3 (*lncFTH1P3*) was overexpressed in PTX-resistant tissue and cells. This promotes PTX resistance through upregulation of *ABCB1* by targeting miR-206 [256]. Yi et al. also demonstrated that miR-381 was downregulated in CIS-resistant tissues and cells, and its overexpression re-sensitized the cells to CIS by targeting *ABCB1* [257].

Chen et al. showed that miR-148/152 family (miR-148a-3p, miR-148b-3p and miR-152-3p) increases sensitivity to DOX. This effect is mediated by targeting *SPIN1*, which regulates drug transporter and metabolizing enzymes ATP Binding Cassette Subfamily B Member 4 (ABCB4)**,** Cytochrome P4502C8 (CYP2C8), UDP Glucuronosyltransferase Family 2 Member B4 (UGT2B4), and UGT2B17 [258].

Some authors have studied the implication of the ABCB9 transporter in drug resistance, which has been described in malignant pleural mesothelioma [259], and non-small cell lung cancer [260], but almost nothing is known in BC. Gong et al. found that miR-24 was downregulated in PTX-resistant BC patients, and its overexpression increased the sensitivity to PTX in resistant cells by targeting *ABCB9* [261].

ATP Binding Cassette Subfamily C Member 1 (ABCC1) is another ABC transporter that carries hydrophobic molecules with neutral or negative charge [250]. Its downregulation by different miRNAs has been found in several BC-resistant cells. Pogribny et al. found that miR-345 and miR-7 were downregulated in CIS-resistant cells compared with the parental cell line. It was found that both miRNAs target the *ABCC1* gene. Hence, the upregulation of those molecules gives, as a result, an increased sensitivity to CIS [262]. Besides, Hong et al. observed that miR-7 expression was higher in patients with pathological complete response treated with PTX plus CAR than in patients with non-pathological complete response. Its higher expression was also associated with higher disease-free survival, thus associating miR-7 with better therapeutic response. Besides, it was demonstrated that miR-7 directly targets *BCL-2* and *ABCC1*, and the upregulation of those molecules promoted sensitivity to CAR and PTX *in vitro* [76]. Moreover, Liang et al. determined that miR-326 was downregulated in an MDR cell line and BC tissues. It was demonstrated that miR-326 targets *ABCC1*, and its upregulation augmented the sensibility to DOX and the ETO [263]. Otherwise, miR-134 was also found to be downregulated in BC cells from cell lines and patients resistant to DOX. Lu et al. demonstrated that this molecule targets *ABCC1* and that its upregulation increases DOX sensitivity [264]. The lncRNAs are also supposed to be implicated in drug resistance and sensitivity. According to Chang et al. linc00518 and MRP1 are upregulated in BC tissues and a MDR cell line. The downregulation of linc00518 was associated with enhanced sensitivity to DOX, vincristine, and PTX. It was demonstrated that linc00518 act as a sponge for miR-199a, thus inhibiting its expression, and miR-199a decrease the expression of *ABCC1* by direct targeting [265].

Zeng et al. found that curcumol induces miR-181b-2-3p, which enhances sensitivity to DOX by directly targeting *ABCC3* [266].

The ABCC4 transporter cannot only transport a wide range of drugs but also endogenous molecules [267]. Hu et al. determined that miR-124-3p was downregulated in DOX-resistant cells and BC tissue compared to normal, while *ABCC4* was upregulated. It was also demonstrated that the overexpression of this miRNA in resistant cell lines downregulates *ABCC4*, thus leading to sensibility to DOX [268].

The ABCC5 transporter can transport different drugs and has a high affinity for the cyclic nucleotide cGMP [269]. Zhu et al. found that downregulation of miR-128 is associated with resistance to chemotherapy and poor survival in BC patients. It was demonstrated that this miRNA is able to downregulate *ABCC5*, and its upregulation led to an increased sensitivity to DOX [93]. Furthermore, miR-128 has been linked to resistance to LET. This miRNA is upregulated in LET-resistant cell lines and directly inhibits the expression of TGF-βRI. Inhibition of miR-128 re-sensitize resistant cells [270].

The ABC transporters described above cannot transport mitoxantrone (MIT) efficiently. By studying cells with resistance to this drug, it was found ABCG2, which is also called MIT-resistance-gene (MXR), BC resistance protein (BCRP), or ABCP (ABC transporter in the placenta), as a possible resistance mechanism [250]. ABCG2 is the target of several miRNAs like miR-181a, miR-328, miR-487, and the miR-302s family (miR-302a/b/c/d), which are downregulated in MIT-resistant BC cells [271–274]. Besides the transport of MIT, ABCG2 is also able to transport DTX and CIS. Takahashi et al. described that miR-27b is upregulated in BC patients. This miRNA targets Ectonucleotide Pyrophosphatase/Phosphodiesterase 1 (*ENPP1*), thus promoting the cell surface localization of ABCG2 and attenuates the chemoresistance to DTX [275]. Besides, miR-106a enhances CIS sensitivity through targeting *ABCG2* [77].

Moreover, it has been described that miR-503 is able to regulate different ABC transporters: ABCB1, ABCC1, and ABCG2. Pan et al. confirmed it and showed that its upregulation increases DOX, TAM, and taxol sensitivity in luminal BC. The authors also demonstrated that this effect is mediated by eukaryotic translation initiation factor 4-γ 1 (eIF4G) [276].

## Discussion

The influence of miRNAs on BC treatments response has been widely proven, and our knowledge about it is constantly increasing. Notwithstanding, it is still necessary to better understand the pathways affected by miRNAs, the interactions between them, and their usefulness as potential predictive and therapeutic tools in combination with current treatments.

Three main issues are pursued to take advantage of the usefulness of miRNAs in overcoming drug resistance. First is the use of miRNAs in BC diagnosis, prediction response to treatments, and patients’ prognosis. Certainly, miRNAs are highly stable molecules that can be detected in tissue samples and body fluids. The last can be obtained by minimally invasive procedures, which is relevant to consider miRNAs as potential biomarkers. However, some limitations must be addressed, such as the limited sensitivity of the current technologies and the need to identify and validate miRNA signatures to predict treatment response in different BC subtypes and therapies. The findings obtained from the currently available studies are still inconsistent, owing to a significant variability in the number of enrolled samples, which is insufficient in many cases, or to the unsettled requirements for patients’ inclusion. Second, there are discrepancies between studies regarding the association between miRNAs and their target genes, possibly due to the specific aims of the researchers. Extensive multicenter studies need to be combined with basic research to better characterize specific miRNAs and their related pathways. Therefore, identifying their multiple targets involved in cancer-related pathways is necessary to improve the use of miRNAs in the clinical context. Third, several drugs that block altered pathways in BC are being tested alone or in combination with classical therapies. However, the therapeutic approaches using miRNAs are mainly in the preclinical stage, and only a few of them are undergoing clinical trials in cancer [277, 278]. miRNA mimics or antagonist are primarily used to promote or to inhibit the miRNA’s effect in cancer, depending on their role as anti-tumor or oncogenic agents. These molecules can be administered directly or through several strategies such as viral vectors or nanoparticles to target cancer cells. Nonetheless, drug delivery methods must be improved to address issues such as off-target effects, poor transfection efficacy, and short compound’s average lifetime.

In conclusion, miRNA-based therapeutic approaches to overcome BC resistance are very promising. Moreover, BC requires more precise and individualized management of the patients due to the heterogeneous nature of the disease. In this context, miRNAs could help to identify tumors with worse prognosis and with different response to a specific therapy. In addition, miRNA-signatures could be used to stratify patients and to design personalized approaches for BC treatment. More efforts are needed to define the most relevant miRNAs, to standardize their detection, and to develop specific and effective signatures and miRNA-based delivery strategies. Altogether, these advances could lead to a relevant change in the management of BC patients and improve diagnosis, prognosis, and overall survival.

## References

[1] Sung H, Ferlay J, Siegel RL, Laversanne M, Soerjomataram I, Jemal A, et al. (2021) Global cancer statistics 2020: GLOBOCAN estimates of incidence and mortality worldwide for 36 cancers in 185 countries. *CA Cancer J Clin*. 10.3322/caac.21660 33538338

[2] Abdalla F, Singh B, Bhat HK. MicroRNAs and gene regulation in breast cancer. *J Biochem Mol Toxicol* 2020; 34(11):e22567. 10.1002/jbt.22567 32729651

[3] Abolghasemi M, Tehrani SS, Yousefi T, Karimian A, Mahmoodpoor A, Ghamari A (2020). MicroRNAs in breast cancer: Roles, functions, and mechanism of actions. Journal of Cellular Physiology.

[4] Dastmalchi N, Safaralizadeh R, Baradaran B, Hosseinpourfeizi M, Baghbanzadeh A. An update review of deregulated tumor suppressive microRNAs and their contribution in various molecular subtypes of breast cancer. *Gene* 2020; 729:144301. 10.1016/j.gene.2019.144301 31884105

[5] Ding L, Gu H, Xiong X, Ao H, Cao J, Lin W, et al. MicroRNAs involved in carcinogenesis, prognosis, therapeutic resistance and applications in human triple-negative breast cancer. *Cells* 2019; 8(12). 10.3390/cells8121492 PMC6953059 31766744

[6] Fridrichova I, Zmetakova I. MicroRNAs contribute to breast cancer invasiveness. *Cells* 2019; 8(11). 10.3390/cells8111361 PMC6912645 31683635

[7] Kandettu A, Radhakrishnan R, Chakrabarty S, Sriharikrishnaa S, Kabekkodu SP. The emerging role of miRNA clusters in breast cancer progression. *Biochim Biophys Acta Rev Cancer* 2020; 1874(2):188413. 10.1016/j.bbcan.2020.188413 32827583

[8] Khalife H, Skafi N, Fayyad-Kazan M, Badran B (2020). MicroRNAs in breast cancer: New maestros defining the melody. Cancer Genetics.

[9] Kudela E, Samec M, Koklesova L, Liskova A, Kubatka P, Kozubik E, et al. miRNA expression profiles in luminal A breast cancer-implications in biology, prognosis, and prediction of response to hormonal treatment. *Int J Mol Sci* 2020; 21(20). 10.3390/ijms21207691 PMC7589921 33080858

[10] Loh HY, Norman BP, Lai KS, Rahman N, Alitheen NBM, Osman MA. The regulatory role of microRNAs in breast cancer. *Int J Mol Sci* 2019; 20(19). 10.3390/ijms20194940 PMC6801796 31590453

[11] Mandujano-Tinoco EA, Garcia-Venzor A, Melendez-Zajgla J, Maldonado V (2018). New emerging roles of microRNAs in breast cancer. Breast Cancer Research and Treatment.

[12] Niu L, Yang W, Duan L, Wang X, Li Y, Xu C (2021). Biological implications and clinical potential of metastasis-related miRNA in colorectal cancer. Mol Ther Nucleic Acids.

[13] Petri BJ, Klinge CM (2020). Regulation of breast cancer metastasis signaling by miRNAs. Cancer and Metastasis Reviews.

[14] Plantamura I, Cosentino G, Cataldo A (2018). MicroRNAs and DNA-damaging drugs in breast cancer: Strength in numbers. Frontiers in Oncology.

[15] Saikia M, Paul S, Chakraborty S. Role of microRNA in forming breast carcinoma. *Life Sci* 2020; 259:118256. 10.1016/j.lfs.2020.118256 32822719

[16] Zhang Y, Xu B, Zhang XP (2018). Effects of miRNAs on functions of breast cancer stem cells and treatment of breast cancer. Oncotargets and Therapy.

[17] Hanahan D, Weinberg RA (2011). Hallmarks of cancer: The next generation. Cell.

[18] Aarts M, Linardopoulos S, Turner NC (2013). Tumour selective targeting of cell cycle kinases for cancer treatment. Current Opinion in Pharmacology.

[19] Diaz-Moralli S, Tarrado-Castellarnau M, Miranda A, Cascante M (2013). Targeting cell cycle regulation in cancer therapy. Pharmacology & Therapeutics.

[20] Otto T, Sicinski P (2017). Cell cycle proteins as promising targets in cancer therapy. Nature Reviews Cancer.

[21] Kastl L, Brown I, Schofield AC (2012). miRNA-34a is associated with docetaxel resistance in human breast cancer cells. Breast Cancer Research and Treatment.

[22] Bao C, Chen J, Chen D, Lu Y, Lou W, Ding B (2020). MiR-93 suppresses tumorigenesis and enhances chemosensitivity of breast cancer via dual targeting E2F1 and CCND1. Cell Death & Disease.

[23] Cataldo A, Cheung DG, Balsari A, Tagliabue E, Coppola V, Iorio MV (2016). miR-302b enhances breast cancer cell sensitivity to cisplatin by regulating E2F1 and the cellular DNA damage response. Oncotarget.

[24] Wang G, Ma C, Shi X, Guo W, Niu J (2019). miR-107 enhances the sensitivity of breast cancer cells to paclitaxel. Open Med (Wars).

[25] Cittelly DM, Das PM, Spoelstra NS, Edgerton SM, Richer JK, Thor AD (2010). Downregulation of miR-342 is associated with tamoxifen resistant breast tumors. Molecular Cancer.

[26] Dou D, Ren X, Han M, Xu X, Ge X, Gu Y (2020). CircUBE2D2 (hsa_circ_0005728) promotes cell proliferation, metastasis and chemoresistance in triple-negative breast cancer by regulating miR-512-3p/CDCA3 axis. Cancer Cell International.

[27] Venturutti L, Cordo Russo RI, Rivas MA, Mercogliano MF, Izzo F, Oakley RH (2016). MiR-16 mediates trastuzumab and lapatinib response in ErbB-2-positive breast and gastric cancer via its novel targets CCNJ and FUBP1. Oncogene.

[28] Yang W, Gu J, Wang X, Wang Y, Feng M, Zhou D (2019). Inhibition of circular RNA CDR1as increases chemosensitivity of 5-FU-resistant BC cells through up-regulating miR-7. Journal of Cellular and Molecular Medicine.

[29] Ichikawa T, Sato F, Terasawa K, Tsuchiya S, Toi M, Tsujimoto G, et al. Trastuzumab produces therapeutic actions by upregulating miR-26a and miR-30b in breast cancer cells. *PLoS One* 2012; 7(2):e31422. 10.1371/journal.pone.0031422 PMC3288043 22384020

[30] Tormo E, Adam-Artigues A, Ballester S, Pineda B, Zazo S, Gonzalez-Alonso P (2017). The role of miR-26a and miR-30b in HER2+ breast cancer trastuzumab resistance and regulation of the CCNE2 gene. Science and Reports.

[31] Liu J, Li X, Wang M, Xiao G, Yang G, Wang H (2018). A miR-26a/E2F7 feedback loop contributes to tamoxifen resistance in ER-positive breast cancer. International Journal of Oncology.

[32] Tormo E, Ballester S, Adam-Artigues A, Burgues O, Alonso E, Bermejo B (2019). The miRNA-449 family mediates doxorubicin resistance in triple-negative breast cancer by regulating cell cycle factors. Science and Reports.

[33] Ward A, Shukla K, Balwierz A, Soons Z, Konig R, Sahin O (2014). MicroRNA-519a is a novel oncomir conferring tamoxifen resistance by targeting a network of tumour-suppressor genes in ER+ breast cancer. The Journal of Pathology.

[34] Wang B, Li D, Filkowski J, Rodriguez-Juarez R, Storozynsky Q, Malach M (2018). A dual role of miR-22 modulated by RelA/p65 in resensitizing fulvestrant-resistant breast cancer cells to fulvestrant by targeting FOXP1 and HDAC4 and constitutive acetylation of p53 at Lys382. Oncogenesis.

[35] Jiang H, Cheng L, Hu P, Liu R (2018). MicroRNA663b mediates TAM resistance in breast cancer by modulating TP73 expression. Molecular Medicine Reports.

[36] Miller TE, Ghoshal K, Ramaswamy B, Roy S, Datta J, Shapiro CL (2008). MicroRNA-221/222 confers tamoxifen resistance in breast cancer by targeting p27Kip1. Journal of Biological Chemistry.

[37] Wang S, Oh DY, Leventaki V, Drakos E, Zhang R, Sahin AA (2019). MicroRNA-17 acts as a tumor chemosensitizer by targeting JAB1/CSN5 in triple-negative breast cancer. Cancer Letters.

[38] Zhang H, Zhao B, Wang X, Zhang F, Yu W (2019). LINC00511 knockdown enhances paclitaxel cytotoxicity in breast cancer via regulating miR-29c/CDK6 axis. Life Sciences.

[39] Zhang W, Jiang H, Chen Y, Ren F (2019). Resveratrol chemosensitizes adriamycin-resistant breast cancer cells by modulating miR-122-5p. Journal of Cellular Biochemistry.

[40] Citron F, Segatto I, Vinciguerra GLR, Musco L, Russo F, Mungo G (2020). Downregulation of miR-223 expression is an early event during mammary transformation and confers resistance to CDK4/6 inhibitors in luminal breast cancer. Cancer Research.

[41] Goodarzi AA, Jeggo PA (2013). The repair and signaling responses to DNA double-strand breaks. Advances in Genetics.

[42] Falck J, Coates J, Jackson SP (2005). Conserved modes of recruitment of ATM, ATR and DNA-PKcs to sites of DNA damage. Nature.

[43] Wu J, Lu LY, Yu X (2010). The role of BRCA1 in DNA damage response. Protein & Cell.

[44] Moskwa P, Buffa FM, Pan Y, Panchakshari R, Gottipati P, Muschel RJ (2011). miR-182-mediated downregulation of BRCA1 impacts DNA repair and sensitivity to PARP inhibitors. Molecular Cell.

[45] He X, Xiao X, Dong L, Wan N, Zhou Z, Deng H (2015). MiR-218 regulates cisplatin chemosensitivity in breast cancer by targeting BRCA1. Tumour Biology.

[46] Xu X, Lv YG, Yan CY, Yi J, Ling R (2016). Enforced expression of hsa-miR-125a-3p in breast cancer cells potentiates docetaxel sensitivity via modulation of BRCA1 signaling. Biochemical and Biophysical Research Communications.

[47] Chen SM, Chou WC, Hu LY, Hsiung CN, Chu HW, Huang YL, et al. The effect of microRNA-124 overexpression on anti-tumor drug sensitivity. *PLoS One* 2015; 10(6):e0128472. 10.1371/journal.pone.0128472 PMC4482746 26115122

[48] Bisso A, Faleschini M, Zampa F, Capaci V, De Santa J, Santarpia L (2013). Oncogenic miR-181a/b affect the DNA damage response in aggressive breast cancer. Cell Cycle.

[49] Xu S, Zhao C, Jia Z, Wang X, Han Y, Yang Z (2017). Down-regulation of PARP1 by miR-891b sensitizes human breast cancer cells to alkylating chemotherapeutic drugs. Archives of Gynecology and Obstetrics.

[50] Mei Z, Su T, Ye J, Yang C, Zhang S, Xie C (2015). The miR-15 family enhances the radiosensitivity of breast cancer cells by targeting G2 checkpoints. Radiation Research.

[51] Lu X, Liu R, Wang M, Kumar AK, Pan F, He L (2020). MicroRNA-140 impedes DNA repair by targeting FEN1 and enhances chemotherapeutic response in breast cancer. Oncogene.

[52] Lin S, Yu L, Song X, Bi J, Jiang L, Wang Y (2019). Intrinsic adriamycin resistance in p53-mutated breast cancer is related to the miR-30c/FANCF/REV1-mediated DNA damage response. Cell Death & Disease.

[53] Bialik S, Zalckvar E, Ber Y, Rubinstein AD, Kimchi A (2010). Systems biology analysis of programmed cell death. Trends in Biochemical Sciences.

[54] Nishida K, Yamaguchi O, Otsu K (2008). Crosstalk between autophagy and apoptosis in heart disease. Circulation Research.

[55] Llambi F, Green DR (2011). Apoptosis and oncogenesis: Give and take in the BCL-2 family. Current Opinion in Genetics & Development.

[56] Benchimol S (2001). p53-dependent pathways of apoptosis. Cell Death and Differentiation.

[57] Bratton SB, Walker G, Srinivasula SM, Sun XM, Butterworth M, Alnemri ES (2001). Recruitment, activation and retention of caspases-9 and -3 by Apaf-1 apoptosome and associated XIAP complexes. EMBO Journal.

[58] Chen S, Rehman SK, Zhang W, Wen A, Yao L, Zhang J (2010). Autophagy is a therapeutic target in anticancer drug resistance. Biochimica et Biophysica Acta.

[59] Hara T, Takamura A, Kishi C, Iemura S, Natsume T, Guan JL (2008). FIP200, a ULK-interacting protein, is required for autophagosome formation in mammalian cells. Journal of Cell Biology.

[60] Lee JT, McCubrey JA (2002). Targeting the Raf kinase cascade in cancer therapy–novel molecular targets and therapeutic strategies. Expert Opinion on Therapeutic Targets.

[61] Ji Y, Di W, Yang Q, Lu Z, Cai W, Wu J (2015). Inhibition of autophagy increases proliferation inhibition and apoptosis induced by the PI3K/mTOR inhibitor NVP-BEZ235 in breast cancer cells. Clinical Laboratory.

[62] McCall K (2010). Genetic control of necrosis - another type of programmed cell death. Current Opinion in Cell Biology.

[63] Kim YS, Morgan MJ, Choksi S, Liu ZG (2007). TNF-induced activation of the Nox1 NADPH oxidase and its role in the induction of necrotic cell death. Molecular Cell.

[64] Strasser A, Vaux DL (2020). Cell death in the origin and treatment of cancer. Molecular Cell.

[65] Zhou M, Liu Z, Zhao Y, Ding Y, Liu H, Xi Y (2010). MicroRNA-125b confers the resistance of breast cancer cells to paclitaxel through suppression of pro-apoptotic Bcl-2 antagonist killer 1 (Bak1) expression. Journal of Biological Chemistry.

[66] Xiang F, Fan Y, Ni Z, Liu Q, Zhu Z, Chen Z (2019). Ursolic acid reverses the chemoresistance of breast cancer cells to paclitaxel by targeting MiRNA-149-5p/MyD88. Frontiers in Oncology.

[67] Cittelly DM, Das PM, Salvo VA, Fonseca JP, Burow ME, Jones FE (2010). Oncogenic HER2{Delta}16 suppresses miR-15a/16 and deregulates BCL-2 to promote endocrine resistance of breast tumors. Carcinogenesis.

[68] O'Brien K, Lowry MC, Corcoran C, Martinez VG, Daly M, Rani S (2015). miR-134 in extracellular vesicles reduces triple-negative breast cancer aggression and increases drug sensitivity. Oncotarget.

[69] Chen L, Bourguignon LY (2014). Hyaluronan-CD44 interaction promotes c-Jun signaling and miRNA21 expression leading to Bcl-2 expression and chemoresistance in breast cancer cells. Molecular Cancer.

[70] Korner C, Keklikoglou I, Bender C, Worner A, Munstermann E, Wiemann S (2013). MicroRNA-31 sensitizes human breast cells to apoptosis by direct targeting of protein kinase C epsilon (PKCepsilon). Journal of Biological Chemistry.

[71] Li ZH, Weng X, Xiong QY, Tu JH, Xiao A, Qiu W (2017). miR-34a expression in human breast cancer is associated with drug resistance. Oncotarget.

[72] Xie X, Hu Y, Xu L, Fu Y, Tu J, Zhao H (2015). The role of miR-125b-mitochondria-caspase-3 pathway in doxorubicin resistance and therapy in human breast cancer. Tumour Biology.

[73] Chen X, Wang YW, Xing AY, Xiang S, Shi DB, Liu L (2016). Suppression of SPIN1-mediated PI3K-Akt pathway by miR-489 increases chemosensitivity in breast cancer. The Journal of Pathology.

[74] Yang G, Wu D, Zhu J, Jiang O, Shi Q, Tian J (2013). Upregulation of miR-195 increases the sensitivity of breast cancer cells to Adriamycin treatment through inhibition of Raf-1. Oncology Reports.

[75] Gu X, Li JY, Guo J, Li PS, Zhang WH (2015). Influence of MiR-451 on drug resistances of paclitaxel-resistant breast cancer cell line. Medical Science Monitor.

[76] Hong T, Ding J, Li W (2019). miR-7 reverses breast cancer resistance to chemotherapy by targeting MRP1 and BCL2. Oncotargets and Therapy.

[77] You F, Luan H, Sun D, Cui T, Ding P, Tang H (2019). miRNA-106a promotes breast cancer cell proliferation, clonogenicity, migration, and invasion through inhibiting apoptosis and chemosensitivity. DNA and Cell Biology.

[78] Manvati S, Mangalhara KC, Kalaiarasan P, Srivastava N, Bamezai RN (2015). miR-24-2 regulates genes in survival pathway and demonstrates potential in reducing cellular viability in combination with docetaxel. Gene.

[79] Long J, Ji Z, Jiang K, Wang Z, Meng G. miR-193b modulates resistance to doxorubicin in human breast cancer cells by downregulating MCL-1. *Biomed Res Int* 2015; 2015:373574. 10.1155/2015/373574 PMC4615858 26526790

[80] Xie Q, Wang S, Zhao Y, Zhang Z, Qin C, Yang X (2017). MiR-519d impedes cisplatin-resistance in breast cancer stem cells by down-regulating the expression of MCL-1. Oncotarget.

[81] Aakko S, Straume AH, Birkeland EE, Chen P, Qiao X, Lonning PE (2019). MYC-induced miR-203b-3p and miR-203a-3p control Bcl-xL expression and paclitaxel sensitivity in tumor cells. Transl Oncol.

[82] Yue J, Lopez JM. Understanding MAPK signaling pathways in apoptosis. *Int J Mol Sci* 2020; 21(7). 10.3390/ijms21072346 PMC7177758 32231094

[83] Mi H, Wang X, Wang F, Li L, Zhu M, Wang N (2018). miR-381 induces sensitivity of breast cancer cells to doxorubicin by inactivation of MAPK signaling via FYN. European Journal of Pharmacology.

[84] Fang Y, Shen H, Cao Y, Li H, Qin R, Chen Q (2014). Involvement of miR-30c in resistance to doxorubicin by regulating YWHAZ in breast cancer cells. Brazilian Journal of Medical and Biological Research.

[85] Han X, Li Q, Liu C, Wang C, Li Y (2019). Overexpression miR-24-3p repressed Bim expression to confer tamoxifen resistance in breast cancer. Journal of Cellular Biochemistry.

[86] Ye Z, Hao R, Cai Y, Wang X, Huang G (2016). Knockdown of miR-221 promotes the cisplatin-inducing apoptosis by targeting the BIM-Bax/Bak axis in breast cancer. Tumour Biology.

[87] Dai H, Xu LY, Qian Q, Zhu QW, Chen WX. MicroRNA-222 promotes drug resistance to doxorubicin in breast cancer via regulation of miR-222/bim pathway. *Biosci Rep* 2019; 39(7). 10.1042/BSR20190650 PMC6629945 31273056

[88] Zheng Y, Lv X, Wang X, Wang B, Shao X, Huang Y (2016). MiR-181b promotes chemoresistance in breast cancer by regulating Bim expression. Oncology Reports.

[89] Zhang Y, He Y, Lu LL, Zhou ZY, Wan NB, Li GP (2019). miRNA-192-5p impacts the sensitivity of breast cancer cells to doxorubicin via targeting peptidylprolyl isomerase A. Kaohsiung Journal of Medical Sciences.

[90] Wang X, Zhu J (2018). Mir-1307 regulates cisplatin resistance by targeting Mdm4 in breast cancer expressing wild type P53. Thorac Cancer.

[91] Sharma S, Nagpal N, Ghosh PC, Kulshreshtha R (2017). P53-miR-191-SOX4 regulatory loop affects apoptosis in breast cancer. RNA.

[92] Kopp F, Oak PS, Wagner E, Roidl A. miR-200c sensitizes breast cancer cells to doxorubicin treatment by decreasing TrkB and Bmi1 expression. *PLoS One* 2012; 7(11):e50469. 10.1371/journal.pone.0050469 PMC3510180 23209748

[93] Zhu Y, Yu F, Jiao Y, Feng J, Tang W, Yao H (2011). Reduced miR-128 in breast tumor-initiating cells induces chemotherapeutic resistance via Bmi-1 and ABCC5. Clinical Cancer Research.

[94] Wu G, Zhou W, Pan X, Sun Y, Xu H, Shi P (2018). miR-100 reverses cisplatin resistance in breast cancer by suppressing HAX-1. Cellular Physiology and Biochemistry.

[95] He H, Tian W, Chen H, Jiang K (2016). MiR-944 functions as a novel oncogene and regulates the chemoresistance in breast cancer. Tumour Biology.

[96] Zhang D, Shi Z, Li M, Mi J. Hypoxia-induced miR-424 decreases tumor sensitivity to chemotherapy by inhibiting apoptosis. *Cell Death Dis* 2014; 5:e1301. 10.1038/cddis.2014.240 PMC4611715 24967963

[97] Tao L, Wu YQ, Zhang SP (2019). MiR-21-5p enhances the progression and paclitaxel resistance in drug-resistant breast cancer cell lines by targeting PDCD4. Neoplasma.

[98] Deng YW, Hao WJ, Li YW, Li YX, Zhao BC, Lu D (2018). Hsa-miRNA-143-3p reverses multidrug resistance of triple-negative breast cancer by inhibiting the expression of its target protein cytokine-induced apoptosis inhibitor 1 *in vivo*. Journal of Breast Cancer.

[99] Duan WJ, Bi PD, Ma Y, Liu NQ, Zhen X (2020). MiR-512-3p regulates malignant tumor behavior and multi-drug resistance in breast cancer cells via targeting Livin. Neoplasma.

[100] Zheng S, Li M, Miao K, Xu H (2020). lncRNA GAS5-promoted apoptosis in triple-negative breast cancer by targeting miR-378a-5p/SUFU signaling. Journal of Cellular Biochemistry.

[101] Yu X, Luo A, Liu Y, Wang S, Li Y, Shi W (2015). MiR-214 increases the sensitivity of breast cancer cells to tamoxifen and fulvestrant through inhibition of autophagy. Molecular Cancer.

[102] Liu ZR, Song Y, Wan LH, Zhang YY, Zhou LM (2016). Over-expression of miR-451a can enhance the sensitivity of breast cancer cells to tamoxifen by regulating 14-3-3zeta, estrogen receptor alpha, and autophagy. Life Sciences.

[103] Lu M, Ding K, Zhang G, Yin M, Yao G, Tian H (2015). MicroRNA-320a sensitizes tamoxifen-resistant breast cancer cells to tamoxifen by targeting ARPP-19 and ERRgamma. Science and Reports.

[104] Ueda S, Takanashi M, Sudo K, Kanekura K, Kuroda M (2020). miR-27a ameliorates chemoresistance of breast cancer cells by disruption of reactive oxygen species homeostasis and impairment of autophagy. Laboratory Investigation.

[105] Han M, Hu J, Lu P, Cao H, Yu C, Li X (2020). Exosome-transmitted miR-567 reverses trastuzumab resistance by inhibiting ATG5 in breast cancer. Cell Death & Disease.

[106] Shi Y, Gong W, Lu L, Wang Y, Ren J. Upregulation of miR-129–5p increases the sensitivity to Taxol through inhibiting HMGB1-mediated cell autophagy in breast cancer MCF-7 cells. *Braz J Med Biol Res* 2019; 52(11):e8657. 10.1590/1414-431X20198657 PMC6826894 31664305

[107] Hsu JL, Hung MC (2016). The role of HER2, EGFR, and other receptor tyrosine kinases in breast cancer. Cancer and Metastasis Reviews.

[108] Yarden Y, Sliwkowski MX (2001). Untangling the ErbB signalling network. Nature Reviews Molecular Cell Biology.

[109] Sergina NV, Moasser MM (2007). The HER family and cancer: Emerging molecular mechanisms and therapeutic targets. Trends in Molecular Medicine.

[110] Gomez GG, Wykosky J, Zanca C, Furnari FB, Cavenee WK (2013). Therapeutic resistance in cancer: MicroRNA regulation of EGFR signaling networks. Cancer Biology & Medicine.

[111] Wang SE, Lin RJ (2013). MicroRNA and HER2-overexpressing cancer. Microrna.

[112] Masuda H, Zhang D, Bartholomeusz C, Doihara H, Hortobagyi GN, Ueno NT (2012). Role of epidermal growth factor receptor in breast cancer. Breast Cancer Research and Treatment.

[113] Huang Q, Wu YY, Xing SJ, Yu ZW (2019). Effect of miR-7 on resistance of breast cancer cells to adriamycin via regulating EGFR/PI3K signaling pathway. European Review for Medical and Pharmacological Sciences.

[114] Li M, Yang J, Zhang L, Tu S, Zhou X, Tan Z (2019). A low-molecular-weight compound exerts anticancer activity against breast and lung cancers by disrupting EGFR/Eps8 complex formation. Journal of Experimental & Clinical Cancer Research.

[115] Ma Y, Bu D, Long J, Chai W, Dong J (2019). LncRNA DSCAM-AS1 acts as a sponge of miR-137 to enhance Tamoxifen resistance in breast cancer. Journal of Cellular Physiology.

[116] He M, Jin Q, Chen C, Liu Y, Ye X, Jiang Y (2019). The miR-186-3p/EREG axis orchestrates tamoxifen resistance and aerobic glycolysis in breast cancer cells. Oncogene.

[117] Corcoran C, Rani S, Breslin S, Gogarty M, Ghobrial IM, Crown J (2014). miR-630 targets IGF1R to regulate response to HER-targeting drugs and overall cancer cell progression in HER2 over-expressing breast cancer. Molecular Cancer.

[118] Ye XM, Zhu HY, Bai WD, Wang T, Wang L, Chen Y (2014). Epigenetic silencing of miR-375 induces trastuzumab resistance in HER2-positive breast cancer by targeting IGF1R. BMC Cancer.

[119] Zhang H, Zheng XD, Zeng XH, Li L, Zhou Q. MiR-520b inhibits IGF-1R to increase doxorubicin sensitivity and promote cell apoptosis in breast cancer. *Yakugaku Zasshi* 2020. 10.1248/yakushi.20-00160 33116033

[120] Oksvold MP, Huitfeldt HS, Langdon WY (2004). Identification of 14-3-3zeta as an EGF receptor interacting protein. FEBS Letters.

[121] Frasor J, Chang EC, Komm B, Lin CY, Vega VB, Liu ET (2006). Gene expression preferentially regulated by tamoxifen in breast cancer cells and correlations with clinical outcome. Cancer Research.

[122] Bergamaschi A, Katzenellenbogen BS (2012). Tamoxifen downregulation of miR-451 increases 14-3-3zeta and promotes breast cancer cell survival and endocrine resistance. Oncogene.

[123] Cooke T, Reeves J, Lanigan A, Stanton P (2001). HER2 as a prognostic and predictive marker for breast cancer. Annals of Oncology.

[124] Pernas S, Tolaney SM (2019). HER2-positive breast cancer: New therapeutic frontiers and overcoming resistance. Ther Adv Med Oncol.

[125] Tan S, Ding K, Chong QY, Zhao J, Liu Y, Shao Y (2017). Post-transcriptional regulation of ERBB2 by miR26a/b and HuR confers resistance to tamoxifen in estrogen receptor-positive breast cancer cells. Journal of Biological Chemistry.

[126] Fang C, Zhao Y, Guo B (2013). MiR-199b-5p targets HER2 in breast cancer cells. Journal of Cellular Biochemistry.

[127] Sajadimajd S, Yazdanparast R, Akram S (2016). Involvement of Numb-mediated HIF-1alpha inhibition in anti-proliferative effect of PNA-antimiR-182 in trastuzumab-sensitive and -resistant SKBR3 cells. Tumour Biology.

[128] Bai WD, Ye XM, Zhang MY, Zhu HY, Xi WJ, Huang X (2014). MiR-200c suppresses TGF-beta signaling and counteracts trastuzumab resistance and metastasis by targeting ZNF217 and ZEB1 in breast cancer. International Journal of Cancer.

[129] Mitra D, Brumlik MJ, Okamgba SU, Zhu Y, Duplessis TT, Parvani JG (2009). An oncogenic isoform of HER2 associated with locally disseminated breast cancer and trastuzumab resistance. Molecular Cancer Therapeutics.

[130] Huynh FC, Jones FE. MicroRNA-7 inhibits multiple oncogenic pathways to suppress HER2Delta16 mediated breast tumorigenesis and reverse trastuzumab resistance. *PLoS One* 2014; 9(12):e114419. 10.1371/journal.pone.0114419 PMC4273950 25532106

[131] Baselga J, Swain SM (2009). Novel anticancer targets: Revisiting ERBB2 and discovering ERBB3. Nature Reviews Cancer.

[132] Bieche I, Onody P, Tozlu S, Driouch K, Vidaud M, Lidereau R (2003). Prognostic value of ERBB family mRNA expression in breast carcinomas. International Journal of Cancer.

[133] Wu H, Zhu S, Mo YY (2009). Suppression of cell growth and invasion by miR-205 in breast cancer. Cell Research.

[134] Iorio MV, Casalini P, Piovan C, Di Leva G, Merlo A, Triulzi T (2009). microRNA-205 regulates HER3 in human breast cancer. Cancer Research.

[135] Cataldo A, Piovan C, Plantamura I, D'Ippolito E, Camelliti S, Casalini P (2018). MiR-205 as predictive biomarker and adjuvant therapeutic tool in combination with trastuzumab. Oncotarget.

[136] Cai Y, Yan X, Zhang G, Zhao W, Jiao S (2016). MicroRNA-205 increases the sensitivity of docetaxel in breast cancer. Oncology Letters.

[137] Lyu H, Huang J, He Z, Liu B (2018). Targeting of HER3 with functional cooperative miRNAs enhances therapeutic activity in HER2-overexpressing breast cancer cells. Biol Proced Online.

[138] De Cola A, Volpe S, Budani MC, Ferracin M, Lattanzio R, Turdo A, et al. miR-205–5p-mediated downregulation of ErbB/HER receptors in breast cancer stem cells results in targeted therapy resistance. *Cell Death Dis* 2015; 6:e1823. 10.1038/cddis.2015.192 PMC4650737 26181203

[139] De Cola A, Lamolinara A, Lanuti P, Rossi C, Iezzi M, Marchisio M (2018). MiR-205-5p inhibition by locked nucleic acids impairs metastatic potential of breast cancer cells. Cell Death & Disease.

[140] Zhao Z, Li R, Sha S, Wang Q, Mao W, Liu T (2014). Targeting HER3 with miR-450b-3p suppresses breast cancer cells proliferation. Cancer Biology & Therapy.

[141] Han G, Qiu N, Luo K, Liang H, Li H. Downregulation of miroRNA-141 mediates acquired resistance to trastuzumab and is associated with poor outcome in breast cancer by upregulating the expression of ERBB4. *J Cell Biochem* 2019. 10.1002/jcb.28416 30746756

[142] Osborne CK, Schiff R (2011). Mechanisms of endocrine resistance in breast cancer. Annual Review of Medicine.

[143] Musgrove EA, Sutherland RL (2009). Biological determinants of endocrine resistance in breast cancer. Nature Reviews Cancer.

[144] Miller TW. Endocrine resistance: What do we know? *Am Soc Clin Oncol Educ Book* 2013. 10.14694/EdBook_AM.2013.33.e37 23714450

[145] Yamashita H, Yando Y, Nishio M, Zhang Z, Hamaguchi M, Mita K (2006). Immunohistochemical evaluation of hormone receptor status for predicting response to endocrine therapy in metastatic breast cancer. Breast Cancer.

[146] Egeland NG, Lunde S, Jonsdottir K, Lende TH, Cronin-Fenton D, Gilje B (2015). The role of microRNAs as predictors of response to tamoxifen treatment in breast cancer patients. International Journal of Molecular Sciences.

[147] Ahmad A, Ginnebaugh KR, Yin S, Bollig-Fischer A, Reddy KB, Sarkar FH (2015). Functional role of miR-10b in tamoxifen resistance of ER-positive breast cancer cells through down-regulation of HDAC4. BMC Cancer.

[148] Leong H, Sloan JR, Nash PD, Greene GL (2005). Recruitment of histone deacetylase 4 to the N-terminal region of estrogen receptor alpha. Molecular Endocrinology.

[149] Liu SS, Li Y, Zhang H, Zhang D, Zhang XB, Wang X (2020). The ERalpha-miR-575-p27 feedback loop regulates tamoxifen sensitivity in ER-positive Breast Cancer. Theranostics.

[150] Manavalan TT, Teng Y, Appana SN, Datta S, Kalbfleisch TS, Li Y (2011). Differential expression of microRNA expression in tamoxifen-sensitive MCF-7 versus tamoxifen-resistant LY2 human breast cancer cells. Cancer Letters.

[151] Wei Y, Lai X, Yu S, Chen S, Ma Y, Zhang Y (2014). Exosomal miR-221/222 enhances tamoxifen resistance in recipient ER-positive breast cancer cells. Breast Cancer Research and Treatment.

[152] Martin EC, Conger AK, Yan TJ, Hoang VT, Miller DF, Buechlein A (2017). MicroRNA-335-5p and -3p synergize to inhibit estrogen receptor alpha expression and promote tamoxifen resistance. FEBS Letters.

[153] He YJ, Wu JZ, Ji MH, Ma T, Qiao EQ, Ma R (2013). miR-342 is associated with estrogen receptor-alpha expression and response to tamoxifen in breast cancer. Experimental and Therapeutic Medicine.

[154] Ljepoja B, Garcia-Roman J, Sommer AK, Wagner E, Roidl A (2019). MiRNA-27a sensitizes breast cancer cells to treatment with selective estrogen receptor modulators. Breast.

[155] Li X, Mertens-Talcott SU, Zhang S, Kim K, Ball J, Safe S (2010). MicroRNA-27a indirectly regulates estrogen receptor alpha expression and hormone responsiveness in MCF-7 breast cancer cells. Endocrinology.

[156] Li Y, Zhou Y, Mao F, Shen S, Zhao B, Xu Y (2020). miR-452 reverses abnormal glycosylation modification of ERalpha and estrogen resistance in TNBC (triple-negative breast cancer) through targeting UGT1A1. Frontiers in Oncology.

[157] Sarkar S, Ghosh A, Banerjee S, Maity G, Das A, Larson MA, et al. CCN5/WISP-2 restores ER- proportional, variant in normal and neoplastic breast cells and sensitizes triple negative breast cancer cells to tamoxifen. *Oncogenesis* 2017; 6(5):e340. 10.1038/oncsis.2017.43 PMC5569333 28530705

[158] Zhang W, Wu M, Chong QY, Zhang M, Zhang X, Hu L, et al. Loss of estrogen-regulated MIR135A1 at 3p21.1 promotes tamoxifen resistance in breast cancer. *Cancer Res* 2018; 78(17):4915–4928. 10.1158/0008-5472.CAN-18-0069 29945962

[159] Osborne CK, Bardou V, Hopp TA, Chamness GC, Hilsenbeck SG, Fuqua SA (2003). Role of the estrogen receptor coactivator AIB1 (SRC-3) and HER-2/neu in tamoxifen resistance in breast cancer. Journal of the National Cancer Institute.

[160] Eedunuri VK, Rajapakshe K, Fiskus W, Geng C, Chew SA, Foley C (2015). miR-137 targets p160 steroid receptor coactivators SRC1, SRC2, and SRC3 and inhibits cell proliferation. Molecular Endocrinology.

[161] Cui J, Yang Y, Li H, Leng Y, Qian K, Huang Q (2015). MiR-873 regulates ERalpha transcriptional activity and tamoxifen resistance via targeting CDK3 in breast cancer cells. Oncogene.

[162] Zhang X, Zhang B, Zhang P, Lian L, Li L, Qiu Z, et al. Norcantharidin regulates ERalpha signaling and tamoxifen resistance via targeting miR-873/CDK3 in breast cancer cells. *PLoS One* 2019; 14(5):e0217181. 10.1371/journal.pone.0217181 PMC6532885 31120927

[163] Miricescu D, Totan A, Stanescu S, II, Badoiu SC, Stefani C, Greabu M. PI3K/AKT/mTOR signaling pathway in breast cancer: From molecular landscape to clinical aspects. *Int J Mol Sci* 2020; 22(1). 10.3390/ijms22010173 PMC7796017 33375317

[164] Alvarez-Garcia V, Tawil Y, Wise HM, Leslie NR (2019). Mechanisms of PTEN loss in cancer: It's all about diversity. Seminars in Cancer Biology.

[165] Liang Z, Li Y, Huang K, Wagar N, Shim H (2011). Regulation of miR-19 to breast cancer chemoresistance through targeting PTEN. Pharmaceutical Research.

[166] Wang ZX, Lu BB, Wang H, Cheng ZX, Yin YM (2011). MicroRNA-21 modulates chemosensitivity of breast cancer cells to doxorubicin by targeting PTEN. Archives of Medical Research.

[167] Gong C, Yao Y, Wang Y, Liu B, Wu W, Chen J (2011). Up-regulation of miR-21 mediates resistance to trastuzumab therapy for breast cancer. Journal of Biological Chemistry.

[168] Yu X, Li R, Shi W, Jiang T, Wang Y, Li C (2016). Silencing of MicroRNA-21 confers the sensitivity to tamoxifen and fulvestrant by enhancing autophagic cell death through inhibition of the PI3K-AKT-mTOR pathway in breast cancer cells. Biomedicine & Pharmacotherapy.

[169] Wu ZH, Tao ZH, Zhang J, Li T, Ni C, Xie J (2016). MiRNA-21 induces epithelial to mesenchymal transition and gemcitabine resistance via the PTEN/AKT pathway in breast cancer. Tumour Biology.

[170] Yu L, Yang Y, Hou J, Zhai C, Song Y, Zhang Z (2015). MicroRNA-144 affects radiotherapy sensitivity by promoting proliferation, migration and invasion of breast cancer cells. Oncology Reports.

[171] Liu T, Guo J, Zhang X (2019). MiR-202-5p/PTEN mediates doxorubicin-resistance of breast cancer cells via PI3K/Akt signaling pathway. Cancer Biology & Therapy.

[172] Gao X, Qin T, Mao J, Zhang J, Fan S, Lu Y (2019). PTENP1/miR-20a/PTEN axis contributes to breast cancer progression by regulating PTEN via PI3K/AKT pathway. Journal of Experimental & Clinical Cancer Research.

[173] Zhong S, Li W, Chen Z, Xu J, Zhao J (2013). MiR-222 and miR-29a contribute to the drug-resistance of breast cancer cells. Gene.

[174] Chen Y, Sun Y, Chen L, Xu X, Zhang X, Wang B (2013). miRNA-200c increases the sensitivity of breast cancer cells to doxorubicin through the suppression of E-cadherin-mediated PTEN/Akt signaling. Molecular Medicine Reports.

[175] Shen H, Wang D, Li L, Yang S, Chen X, Zhou S (2017). MiR-222 promotes drug-resistance of breast cancer cells to adriamycin via modulation of PTEN/Akt/FOXO1 pathway. Gene.

[176] Gu J, Wang Y, Wang X, Zhou D, Shao C, Zhou M (2018). Downregulation of lncRNA GAS5 confers tamoxifen resistance by activating miR-222 in breast cancer. Cancer Letters.

[177] Geng W, Song H, Zhao Q, Dong K, Pu Q, Gao H (2020). miR-520h stimulates drug resistance to paclitaxel by targeting the OTUD3-PTEN axis in breast cancer. BioMed Research International.

[178] Sachdeva M, Wu H, Ru P, Hwang L, Trieu V, Mo YY (2011). MicroRNA-101-mediated Akt activation and estrogen-independent growth. Oncogene.

[179] Haga CL, Velagapudi SP, Strivelli JR, Yang WY, Disney MD, Phinney DG (2015). Small molecule inhibition of miR-544 biogenesis disrupts adaptive responses to hypoxia by modulating ATM-mTOR signaling. ACS Chemical Biology.

[180] Zhang B, Zhao R, He Y, Fu X, Fu L, Zhu Z (2016). MicroRNA 100 sensitizes luminal A breast cancer cells to paclitaxel treatment in part by targeting mTOR. Oncotarget.

[181] Ma T, Yang L, Zhang J (2015). MiRNA5423p downregulation promotes trastuzumab resistance in breast cancer cells via AKT activation. Oncology Reports.

[182] Fu R, Tong JS (2020). miR-126 reduces trastuzumab resistance by targeting PIK3R2 and regulating AKT/mTOR pathway in breast cancer cells. Journal of Cellular and Molecular Medicine.

[183] Pan X, Hong X, Lai J, Cheng L, Cheng Y, Yao M (2020). Exosomal microRNA-221-3p confers adriamycin resistance in breast cancer cells by targeting PIK3R1. Frontiers in Oncology.

[184] Vilquin P, Donini CF, Villedieu M, Grisard E, Corbo L, Bachelot T (2015). MicroRNA-125b upregulation confers aromatase inhibitor resistance and is a novel marker of poor prognosis in breast cancer. Breast Cancer Research.

[185] Dave B, Mittal V, Tan NM, Chang JC (2012). Epithelial-mesenchymal transition, cancer stem cells and treatment resistance. Breast Cancer Research.

[186] Shibue T, Weinberg RA (2017). EMT, CSCs, and drug resistance: The mechanistic link and clinical implications. Nature Reviews. Clinical Oncology.

[187] Smalley M, Piggott L, Clarkson R (2013). Breast cancer stem cells: Obstacles to therapy. Cancer Letters.

[188] Mallini P, Lennard T, Kirby J, Meeson A (2014). Epithelial-to-mesenchymal transition: What is the impact on breast cancer stem cells and drug resistance. Cancer Treatment Reviews.

[189] Zhou S, Schuetz JD, Bunting KD, Colapietro AM, Sampath J, Morris JJ (2001). The ABC transporter Bcrp1/ABCG2 is expressed in a wide variety of stem cells and is a molecular determinant of the side-population phenotype. Nature Medicine.

[190] Dean M, Fojo T, Bates S (2005). Tumour stem cells and drug resistance. Nature Reviews Cancer.

[191] Li X, Lewis MT, Huang J, Gutierrez C, Osborne CK, Wu MF (2008). Intrinsic resistance of tumorigenic breast cancer cells to chemotherapy. Journal of the National Cancer Institute.

[192] Mani SA, Guo W, Liao MJ, Eaton EN, Ayyanan A, Zhou AY (2008). The epithelial-mesenchymal transition generates cells with properties of stem cells. Cell.

[193] Pinto CA, Widodo E, Waltham M, Thompson EW (2013). Breast cancer stem cells and epithelial mesenchymal plasticity - Implications for chemoresistance. Cancer Letters.

[194] Ansieau S (2013). EMT in breast cancer stem cell generation. Cancer Letters.

[195] De Craene B, Berx G (2013). Regulatory networks defining EMT during cancer initiation and progression. Nature Reviews Cancer.

[196] Brabletz S, Bajdak K, Meidhof S, Burk U, Niedermann G, Firat E (2011). The ZEB1/miR-200 feedback loop controls Notch signalling in cancer cells. EMBO Journal.

[197] Burk U, Schubert J, Wellner U, Schmalhofer O, Vincan E, Spaderna S (2008). A reciprocal repression between ZEB1 and members of the miR-200 family promotes EMT and invasion in cancer cells. EMBO Reports.

[198] Gregory PA, Bert AG, Paterson EL, Barry SC, Tsykin A, Farshid G (2008). The miR-200 family and miR-205 regulate epithelial to mesenchymal transition by targeting ZEB1 and SIP1. Nature Cell Biology.

[199] Hurteau GJ, Carlson JA, Roos E, Brock GJ (2009). Stable expression of miR-200c alone is sufficient to regulate TCF8 (ZEB1) and restore E-cadherin expression. Cell Cycle.

[200] Cochrane DR, Spoelstra NS, Howe EN, Nordeen SK, Richer JK (2009). MicroRNA-200c mitigates invasiveness and restores sensitivity to microtubule-targeting chemotherapeutic agents. Molecular Cancer Therapeutics.

[201] Yang X, Hu Q, Hu LX, Lin XR, Liu JQ, Lin X (2017). miR-200b regulates epithelial-mesenchymal transition of chemo-resistant breast cancer cells by targeting FN1. Discovery Medicine.

[202] Knezevic J, Pfefferle AD, Petrovic I, Greene SB, Perou CM, Rosen JM (2015). Expression of miR-200c in claudin-low breast cancer alters stem cell functionality, enhances chemosensitivity and reduces metastatic potential. Oncogene.

[203] Li CY, Miao KL, Chen Y, Liu LY, Zhao GB, Lin MH (2018). Jagged2 promotes cancer stem cell properties of triple negative breast cancer cells and paclitaxel resistance via regulating microRNA-200. European Review for Medical and Pharmacological Sciences.

[204] Chen J, Tian W, He H, Chen F, Huang J, Wang X (2018). Downregulation of miR200c3p contributes to the resistance of breast cancer cells to paclitaxel by targeting SOX2. Oncology Reports.

[205] Soung YH, Chung H, Yan C, Ju J, Chung J. Arrestin domain containing 3 reverses epithelial to mesenchymal transition and chemo-resistance of TNBC cells by up-regulating expression of miR-200b. *Cells* 2019; 8(7). 10.3390/cells8070692 PMC6679179 31295851

[206] Manavalan TT, Teng Y, Litchfield LM, Muluhngwi P, Al-Rayyan N, Klinge CM. Reduced expression of miR-200 family members contributes to antiestrogen resistance in LY2 human breast cancer cells. *PLoS One* 2013; 8(4):e62334. 10.1371/journal.pone.0062334 PMC3633860 23626803

[207] Gao Y, Zhang W, Liu C, Li G (2019). miR-200 affects tamoxifen resistance in breast cancer cells through regulation of MYB. Science and Reports.

[208] Tang H, Song C, Ye F, Gao G, Ou X, Zhang L (2019). miR-200c suppresses stemness and increases cellular sensitivity to trastuzumab in HER2+ breast cancer. Journal of Cellular and Molecular Medicine.

[209] Song W, Wu S, Wu Q, Zhou L, Yu L, Zhu B (2019). The microRNA-141-3p/ CDK8 pathway regulates the chemosensitivity of breast cancer cells to trastuzumab. Journal of Cellular Biochemistry.

[210] Chen J, Tian W, Cai H, He H, Deng Y (2012). Down-regulation of microRNA-200c is associated with drug resistance in human breast cancer. Medical Oncology.

[211] Lee JW, Guan W, Han S, Hong DK, Kim LS, Kim H (2018). MicroRNA-708-3p mediates metastasis and chemoresistance through inhibition of epithelial-to-mesenchymal transition in breast cancer. Cancer Science.

[212] Wang G, Dong Y, Liu H, Ji N, Cao J, Liu A (2019). Loss of miR-873 contributes to gemcitabine resistance in triple-negative breast cancer via targeting ZEB1. Oncology Letters.

[213] Gao L, Guo Q, Li X, Yang X, Ni H, Wang T (2019). MiR-873/PD-L1 axis regulates the stemness of breast cancer cells. eBioMedicine.

[214] Bockhorn J, Dalton R, Nwachukwu C, Huang S, Prat A, Yee K (2013). MicroRNA-30c inhibits human breast tumour chemotherapy resistance by regulating TWF1 and IL-11. Nature Communications.

[215] Guan X, Gu S, Yuan M, Zheng X, Wu J (2019). MicroRNA-33a-5p overexpression sensitizes triple-negative breast cancer to doxorubicin by inhibiting eIF5A2 and epithelial-mesenchymal transition. Oncology Letters.

[216] Luan QX, Zhang BG, Li XJ, Guo MY (2016). MiR-129-5p is downregulated in breast cancer cells partly due to promoter H3K27m3 modification and regulates epithelial-mesenchymal transition and multi-drug resistance. European Review for Medical and Pharmacological Sciences.

[217] Yao N, Fu Y, Chen L, Liu Z, He J, Zhu Y (2019). Long non-coding RNA NONHSAT101069 promotes epirubicin resistance, migration, and invasion of breast cancer cells through NONHSAT101069/miR-129-5p/Twist1 axis. Oncogene.

[218] Du F, Yu L, Wu Y, Wang S, Yao J, Zheng X (2019). miR-137 alleviates doxorubicin resistance in breast cancer through inhibition of epithelial-mesenchymal transition by targeting DUSP4. Cell Death & Disease.

[219] Yan L, Yang S, Yue CX, Wei XY, Peng W, Dong ZY (2020). Long noncoding RNA H19 acts as a miR-340-3p sponge to promote epithelial-mesenchymal transition by regulating YWHAZ expression in paclitaxel-resistant breast cancer cells. Environmental Toxicology.

[220] Shi S, Chen X, Liu H, Yu K, Bao Y, Chai J (2019). LGR5 acts as a target of miR-340-5p in the suppression of cell progression and drug resistance in breast cancer via Wnt/beta-catenin pathway. Gene.

[221] Jiang L, He D, Yang D, Chen Z, Pan Q, Mao A (2014). MiR-489 regulates chemoresistance in breast cancer via epithelial mesenchymal transition pathway. FEBS Letters.

[222] Dong H, Hu J, Zou K, Ye M, Chen Y, Wu C (2019). Activation of LncRNA TINCR by H3K27 acetylation promotes trastuzumab resistance and epithelial-mesenchymal transition by targeting microRNA-125b in breast cancer. Molecular Cancer.

[223] Hu SH, Wang CH, Huang ZJ, Liu F, Xu CW, Li XL (2016). miR-760 mediates chemoresistance through inhibition of epithelial mesenchymal transition in breast cancer cells. European Review for Medical and Pharmacological Sciences.

[224] Fu H, Fu L, Xie C, Zuo WS, Liu YS, Zheng MZ (2017). miR-375 inhibits cancer stem cell phenotype and tamoxifen resistance by degrading HOXB3 in human ER-positive breast cancer. Oncology Reports.

[225] Ward A, Balwierz A, Zhang JD, Kublbeck M, Pawitan Y, Hielscher T (2013). Re-expression of microRNA-375 reverses both tamoxifen resistance and accompanying EMT-like properties in breast cancer. Oncogene.

[226] Zhang HD, Sun DW, Mao L, Zhang J, Jiang LH, Li J (2015). MiR-139-5p inhibits the biological function of breast cancer cells by targeting Notch1 and mediates chemosensitivity to docetaxel. Biochemical and Biophysical Research Communications.

[227] Li XJ, Ji MH, Zhong SL, Zha QB, Xu JJ, Zhao JH (2012). MicroRNA-34a modulates chemosensitivity of breast cancer cells to adriamycin by targeting Notch1. Archives of Medical Research.

[228] Kang L, Mao J, Tao Y, Song B, Ma W, Lu Y (2015). MicroRNA-34a suppresses the breast cancer stem cell-like characteristics by downregulating Notch1 pathway. Cancer Science.

[229] Gong LG, Shi JC, Shang J, Hao JG, Du X (2019). Effect of miR-34a on resistance to sunitinib in breast cancer by regulating the Wnt/beta-catenin signaling pathway. European Review for Medical and Pharmacological Sciences.

[230] Palyi-Krekk Z, Barok M, Isola J, Tammi M, Szollosi J, Nagy P (2007). Hyaluronan-induced masking of ErbB2 and CD44-enhanced trastuzumab internalisation in trastuzumab resistant breast cancer. European Journal of Cancer.

[231] Boulbes DR, Chauhan GB, Jin Q, Bartholomeusz C, Esteva FJ (2015). CD44 expression contributes to trastuzumab resistance in HER2-positive breast cancer cells. Breast Cancer Research and Treatment.

[232] Liu C, Xing H, Guo C, Yang Z, Wang Y, Wang Y (2019). MiR-124 reversed the doxorubicin resistance of breast cancer stem cells through STAT3/HIF-1 signaling pathways. Cell Cycle.

[233] Tan W, Tang H, Jiang X, Ye F, Huang L, Shi D (2019). Metformin mediates induction of miR-708 to inhibit self-renewal and chemoresistance of breast cancer stem cells through targeting CD47. Journal of Cellular and Molecular Medicine.

[234] Zhou Y, Hu Y, Yang M, Jat P, Li K, Lombardo Y (2014). The miR-106b~25 cluster promotes bypass of doxorubicin-induced senescence and increase in motility and invasion by targeting the E-cadherin transcriptional activator EP300. Cell Death and Differentiation.

[235] Hu Y, Li K, Asaduzzaman M, Cuella R, Shi H, Raguz S (2016). MiR-106b~25 cluster regulates multidrug resistance in an ABC transporter-independent manner via downregulation of EP300. Oncology Reports.

[236] Guarnieri AL, Towers CG, Drasin DJ, Oliphant MUJ, Andrysik Z, Hotz TJ (2018). The miR-106b-25 cluster mediates breast tumor initiation through activation of NOTCH1 via direct repression of NEDD4L. Oncogene.

[237] Li HY, Liang JL, Kuo YL, Lee HH, Calkins MJ, Chang HT (2017). miR-105/93-3p promotes chemoresistance and circulating miR-105/93-3p acts as a diagnostic biomarker for triple negative breast cancer. Breast Cancer Research.

[238] Chen Z, Pan T, Jiang D, Jin L, Geng Y, Feng X (2020). The lncRNA-GAS5/miR-221-3p/DKK2 axis modulates ABCB1-mediated adriamycin resistance of breast cancer via the Wnt/beta-catenin signaling pathway. Mol Ther Nucleic Acids.

[239] Tang T, Cheng Y, She Q, Jiang Y, Chen Y, Yang W (2018). Long non-coding RNA TUG1 sponges miR-197 to enhance cisplatin sensitivity in triple negative breast cancer. Biomedicine & Pharmacotherapy.

[240] Wu D, Zhang J, Lu Y, Bo S, Li L, Wang L (2019). miR-140-5p inhibits the proliferation and enhances the efficacy of doxorubicin to breast cancer stem cells by targeting Wnt1. Cancer Gene Therapy.

[241] Yu Y, Yin W, Yu ZH, Zhou YJ, Chi JR, Ge J (2019). miR-190 enhances endocrine therapy sensitivity by regulating SOX9 expression in breast cancer. Journal of Experimental & Clinical Cancer Research.

[242] Jia Z, Zhu H, Sun H, Hua Y, Zhang G, Jiang J (2020). Adipose mesenchymal stem cell-derived exosomal microRNA-1236 reduces resistance of breast cancer cells to cisplatin by suppressing SLC9A1 and the Wnt/beta-catenin signaling. Cancer Manag Res.

[243] Liang Y, Song X, Li Y, Su P, Han D, Ma T (2019). circKDM4C suppresses tumor progression and attenuates doxorubicin resistance by regulating miR-548p/PBLD axis in breast cancer. Oncogene.

[244] Wang YY, Yan L, Yang S, Xu HN, Chen TT, Dong ZY, et al. Long noncoding RNA AC073284.4 suppresses epithelial-mesenchymal transition by sponging miR-18b-5p in paclitaxel-resistant breast cancer cells. *J Cell Physiol* 2019; 234(12):23202–23215. 10.1002/jcp.28887 31215650

[245] Santos JC, Lima NDS, Sarian LO, Matheu A, Ribeiro ML, Derchain SFM (2018). Exosome-mediated breast cancer chemoresistance via miR-155 transfer. Science and Reports.

[246] Yang LW, Wu XJ, Liang Y, Ye GQ, Che YC, Wu XZ (2020). miR-155 increases stemness and decitabine resistance in triple-negative breast cancer cells by inhibiting TSPAN5. Molecular Carcinogenesis.

[247] Chu S, Liu G, Xia P, Chen G, Shi F, Yi T (2017). miR-93 and PTEN: Key regulators of doxorubicin-resistance and EMT in breast cancer. Oncology Reports.

[248] Duan X, Liu X, Cao Y, Li Y, Silayiding A, Zhang L (2021). Effect of microRNA-766 promotes proliferation, chemoresistance, migration, and invasion of breast cancer cells. Clinical Breast Cancer.

[249] De Mattos-Arruda L, Bottai G, Nuciforo PG, Di Tommaso L, Giovannetti E, Peg V (2015). MicroRNA-21 links epithelial-to-mesenchymal transition and inflammatory signals to confer resistance to neoadjuvant trastuzumab and chemotherapy in HER2-positive breast cancer patients. Oncotarget.

[250] Gottesman MM, Fojo T, Bates SE (2002). Multidrug resistance in cancer: Role of ATP-dependent transporters. Nature Reviews Cancer.

[251] Zhu X, Li Y, Shen H, Li H, Long L, Hui L (2013). miR-137 restoration sensitizes multidrug-resistant MCF-7/ADM cells to anticancer agents by targeting YB-1. Acta Biochimica et Biophysica Sinica (Shanghai).

[252] Bao L, Hazari S, Mehra S, Kaushal D, Moroz K, Dash S (2012). Increased expression of P-glycoprotein and doxorubicin chemoresistance of metastatic breast cancer is regulated by miR-298. American Journal of Pathology.

[253] Kovalchuk O, Filkowski J, Meservy J, Ilnytskyy Y, Tryndyak VP, Chekhun VF (2008). Involvement of microRNA-451 in resistance of the MCF-7 breast cancer cells to chemotherapeutic drug doxorubicin. Molecular Cancer Therapeutics.

[254] Thorne JL, Battaglia S, Baxter DE, Hayes JL, Hutchinson SA, Jana S (2018). MiR-19b non-canonical binding is directed by HuR and confers chemosensitivity through regulation of P-glycoprotein in breast cancer. Biochimica et Biophysica Acta, Gene Regulatory Mechanisms.

[255] He DX, Gu XT, Jiang L, Jin J, Ma X (2014). A methylation-based regulatory network for microRNA 320a in chemoresistant breast cancer. Molecular Pharmacology.

[256] Wang R, Zhang T, Yang Z, Jiang C, Seng J (2018). Long non-coding RNA FTH1P3 activates paclitaxel resistance in breast cancer through miR-206/ABCB1. Journal of Cellular and Molecular Medicine.

[257] Yi D, Xu L, Wang R, Lu X, Sang J (2019). miR-381 overcomes cisplatin resistance in breast cancer by targeting MDR1. Cell Biology International.

[258] Chen X, Wang YW, Gao P (2018). SPIN1, negatively regulated by miR-148/152, enhances Adriamycin resistance via upregulating drug metabolizing enzymes and transporter in breast cancer. Journal of Experimental & Clinical Cancer Research.

[259] Moody HL, Lind MJ, Maher SG (2017). MicroRNA-31 regulates chemosensitivity in malignant pleural mesothelioma. Mol Ther Nucleic Acids.

[260] Dong Z, Zhong Z, Yang L, Wang S, Gong Z (2014). MicroRNA-31 inhibits cisplatin-induced apoptosis in non-small cell lung cancer cells by regulating the drug transporter ABCB9. Cancer Letters.

[261] Gong JP, Yang L, Tang JW, Sun P, Hu Q, Qin JW (2016). Overexpression of microRNA-24 increases the sensitivity to paclitaxel in drug-resistant breast carcinoma cell lines via targeting ABCB9. Oncology Letters.

[262] Pogribny IP, Filkowski JN, Tryndyak VP, Golubov A, Shpyleva SI, Kovalchuk O (2010). Alterations of microRNAs and their targets are associated with acquired resistance of MCF-7 breast cancer cells to cisplatin. International Journal of Cancer.

[263] Liang Z, Wu H, Xia J, Li Y, Zhang Y, Huang K (2010). Involvement of miR-326 in chemotherapy resistance of breast cancer through modulating expression of multidrug resistance-associated protein 1. Biochemical Pharmacology.

[264] Lu L, Ju F, Zhao H, Ma X (2015). MicroRNA-134 modulates resistance to doxorubicin in human breast cancer cells by downregulating ABCC1. Biotechnology Letters.

[265] Chang L, Hu Z, Zhou Z, Zhang H (2018). Linc00518 contributes to multidrug resistance through regulating the MiR-199a/MRP1 axis in breast cancer. Cellular Physiology and Biochemistry.

[266] Zeng C, Fan D, Xu Y, Li X, Yuan J, Yang Q, et al. Curcumol enhances the sensitivity of doxorubicin in triple-negative breast cancer via regulating the miR-181b-2–3p-ABCC3 axis. *Biochem Pharmacol* 2020; 174:113795. 10.1016/j.bcp.2020.113795 31926937

[267] Russel FG, Koenderink JB, Masereeuw R (2008). Multidrug resistance protein 4 (MRP4/ABCC4): A versatile efflux transporter for drugs and signalling molecules. Trends in Pharmacological Sciences.

[268] Hu D, Li M, Su J, Miao K, Qiu X (2019). Dual-targeting of miR-124-3p and ABCC4 promotes sensitivity to adriamycin in breast cancer cells. Genetic Testing and Molecular Biomarkers.

[269] Jedlitschky G, Burchell B, Keppler D (2000). The multidrug resistance protein 5 functions as an ATP-dependent export pump for cyclic nucleotides. Journal of Biological Chemistry.

[270] Masri S, Liu Z, Phung S, Wang E, Yuan YC, Chen S (2010). The role of microRNA-128a in regulating TGFbeta signaling in letrozole-resistant breast cancer cells. Breast Cancer Research and Treatment.

[271] Jiao X, Zhao L, Ma M, Bai X, He M, Yan Y (2013). MiR-181a enhances drug sensitivity in mitoxantone-resistant breast cancer cells by targeting breast cancer resistance protein (BCRP/ABCG2). Breast Cancer Research and Treatment.

[272] Pan YZ, Morris ME, Yu AM (2009). MicroRNA-328 negatively regulates the expression of breast cancer resistance protein (BCRP/ABCG2) in human cancer cells. Molecular Pharmacology.

[273] Ma MT, He M, Wang Y, Jiao XY, Zhao L, Bai XF (2013). MiR-487a resensitizes mitoxantrone (MX)-resistant breast cancer cells (MCF-7/MX) to MX by targeting breast cancer resistance protein (BCRP/ABCG2). Cancer Letters.

[274] Wang Y, Zhao L, Xiao Q, Jiang L, He M, Bai X (2016). miR-302a/b/c/d cooperatively inhibit BCRP expression to increase drug sensitivity in breast cancer cells. Gynecologic Oncology.

[275] Takahashi RU, Miyazaki H, Takeshita F, Yamamoto Y, Minoura K, Ono M (2015). Loss of microRNA-27b contributes to breast cancer stem cell generation by activating ENPP1. Nature Communications.

[276] Pan X, Yang X, Zang J, Zhang S, Huang N, Guan X (2017). Downregulation of eIF4G by microRNA-503 enhances drug sensitivity of MCF-7/ADR cells through suppressing the expression of ABC transport proteins. Oncology Letters.

[277] Smolle MA, Calin HN, Pichler M, Calin GA (2017). Noncoding RNAs and immune checkpoints-clinical implications as cancer therapeutics. FEBS Journal.

[278] Rupaimoole R, Slack FJ (2017). MicroRNA therapeutics: Towards a new era for the management of cancer and other diseases. Nature Reviews. Drug Discovery.

